# METTL3 confers oxaliplatin resistance through the activation of G6PD-enhanced pentose phosphate pathway in hepatocellular carcinoma

**DOI:** 10.1038/s41418-024-01406-2

**Published:** 2024-10-29

**Authors:** Xiaohan Jin, Yongrui Lv, Fengjie Bie, Jinling Duan, Chao Ma, Miaomiao Dai, Jiewei Chen, Lianghe Lu, Shuidan Xu, Jie Zhou, Si Li, Jiong Bi, Fengwei Wang, Dan Xie, Muyan Cai

**Affiliations:** 1 State Key Laboratory of Oncology in South China, Guangdong Provincial Clinical Research Center for Cancer, Sun Yat-sen University Cancer Center, Guangzhou, PR China; 2 State Key Laboratory of Respiratory Disease, Institute of Pulmonary Diseases, Department of Oncology, Guangzhou Chest Hospital, Guangzhou Medical University, Guangzhou, PR China; 3 Breast Disease Specialist Hospital of Guangdong Provincial Hospital of Chinese Medicine, Guangdong Provincial Hospital of Chinese Medicine, Guangzhou, PR China; 4 Department of Liver Surgery, Sun Yat-sen University Cancer Center, Guangzhou, China; 5 Laboratory of General Surgery, The First Affiliated Hospital, Sun Yat-sen University, Guangzhou, China

**Keywords:** Cancer metabolism, Epigenetics

## Abstract

Oxaliplatin-based therapeutics is a widely used treatment approach for hepatocellular carcinoma (HCC) patients; however, drug resistance poses a significant clinical challenge. Epigenetic modifications have been implicated in the development of drug resistance. In our study, employing siRNA library screening, we identified that silencing the m^6^A writer METTL3 significantly enhanced the sensitivity to oxaliplatin in both in vivo and in vitro HCC models. Further investigations through combined RNA-seq and non-targeted metabolomics analysis revealed that silencing METTL3 impeded the pentose phosphate pathway (PPP), leading to a reduction in NADPH and nucleotide precursors. This disruption induced DNA damage, decreased DNA synthesis, and ultimately resulted in cell cycle arrest. Mechanistically, METTL3 was found to modify E3 ligase TRIM21 near the 3’UTR with N^6^-methyladenosine, leading to reduced RNA stability upon recognition by YTHDF2. TRIM21, in turn, facilitated the degradation of the rate-limiting enzyme of PPP, G6PD, through the ubiquitination-proteasome pathway. Importantly, high expression of METTL3 was significantly associated with adverse prognosis and oxaliplatin resistance in HCC patients. Notably, treatment with the specific METTL3 inhibitor, STM2457, significantly improved the efficacy of oxaliplatin. These findings underscore the critical role of the METTL3/TRIM21/G6PD axis in driving oxaliplatin resistance and present a promising strategy to overcome chemoresistance in HCC.

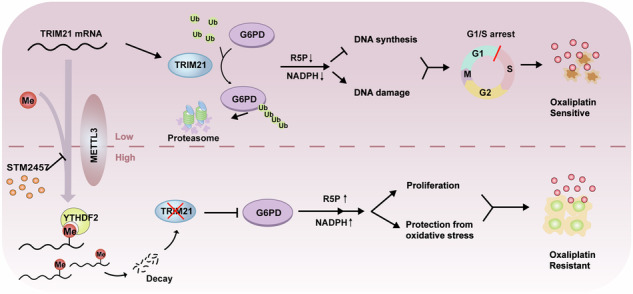

## Introduction

Hepatocellular carcinoma (HCC) is an aggressive malignant tumor with a poor prognosis and the third-most-common cause of cancer-related mortality worldwide [1]. Chemotherapy has been a major therapeutic approach for HCC treatment, which can be delivered via trans-arterial chemoembolization (TACE) or hepatic arterial infusion (HAIC) [2]. Oxaliplatin (OXA)-based HAIC is considered one of the preferred chemotherapeutic strategies for patients who have undergone surgical resection and is used as maintenance therapy for advanced HCC [3–5]. However, the de novo or acquired resistance to OXA-based therapy remains a major challenge, leading to treatment failure and dismal clinical outcomes. There is an urgent need to explore mechanisms of resistance to OXA-based therapy and identify a novel therapeutic strategy to enhance the effectiveness of OXA treatment in HCC.

Recent studies have demonstrated that OXA resistance is associated with various biological processes, including epigenetic modification, DNA repair, cell metabolism, and apoptosis [6, 7]. Although a genetic basis for drug resistance can contribute to the failure of anticancer therapy, numerous observations imply epigenetic modification plays a crucial role in drug non-responsiveness without gene mutations [8–10]. There is increasing evidence that various types of epigenetic modifications can impact almost all aspects of RNA metabolism, including stability, splicing, localization, and translation [11]. Dysregulation of these modifications can lead to drug resistance and hinder treatment efforts [12, 13]. Our recent study implies the role of m^6^A modification in circRNA translation and its contribution to cisplatin chemoresistance in HCC [14]. Long non‐coding RNA and protein arginine methylation have been found to regulate OXA resistance in HCC by reducing apoptosis and intracellular reactive oxygen [2, 15]. Despite the complex nature of OXA resistance mechanisms, identifying intervention targets based on downstream pathways remains an important avenue for advancing HCC treatment.

In the current study, we performed an in vitro screening with an epigenetics-related RNA interference library to explore the potential regulators of OXA resistance in three HCC cell lines. Notably, we identified methyltransferase-like 3 (METTL3), a key component of the m^6^A methylase complex, as the crucial gene conferring OXA resistance in HCC, which was verified by in vitro and in vivo studies. In addition, high expression of METTL3 was significantly correlated with the adverse prognosis and poor OXA therapeutic efficacy in HCC patients. More importantly, we found that the specific METTL3 inhibitor, STM2457, dramatically enhanced the efficacy of OXA management. Mechanistically, METTL3 down-regulated the expression of E3 ligase TRIM21 via m^6^A modification to increase the protein stability of glucose-6-phosphate dehydrogenase (G6PD), contributing to reduced HCC sensitivity to OXA. Thus, our findings present the vital role of the METTL3/TRIM21/G6PD axis for driving drug resistance and pave the way for creating a promising strategy to overcome oxaliplatin resistance in HCC.

## Results

### METTL3 is involved in oxaliplatin resistance in HCC

**Fig. 1:**
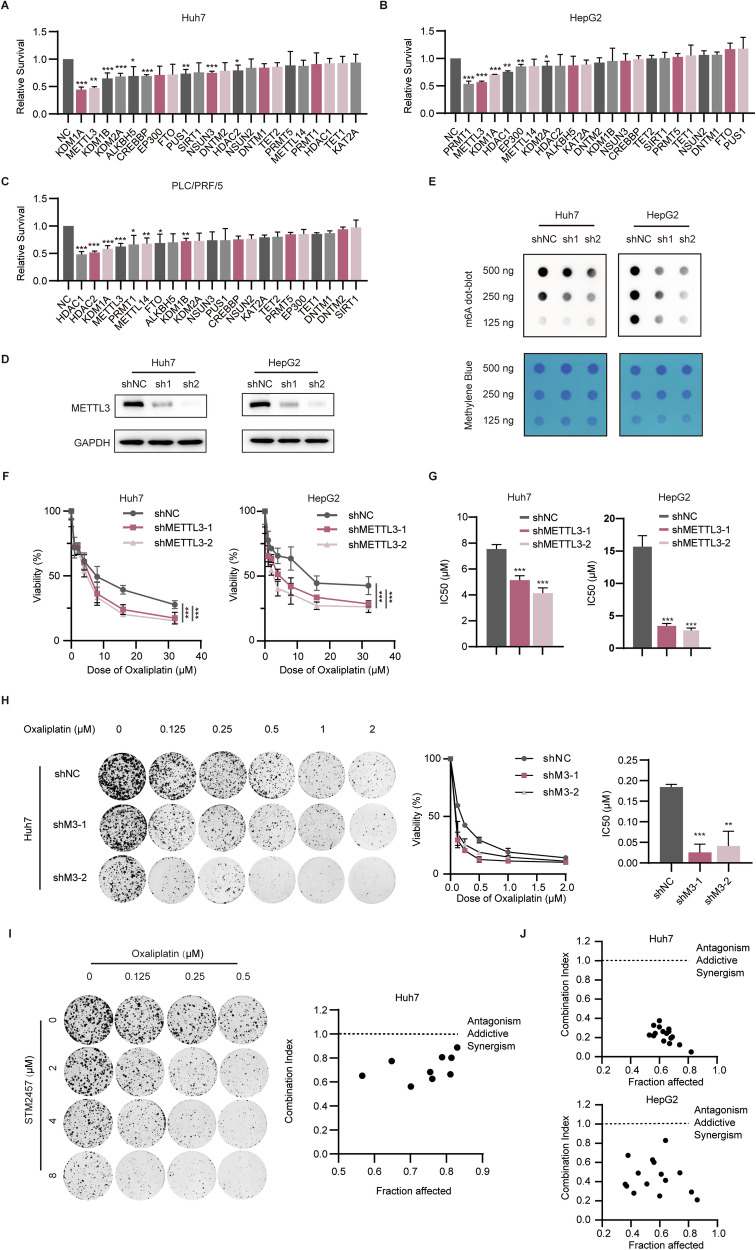
METTL3 is involved in oxaliplatin resistance in HCC. **A**–**C** Relative survival rate of Huh7, HepG2, and PLC/PRF/5 cells with specific siRNA transfection following OXA treatment for 48 h. **D** The efficiency of METTL3 knockdown verified at protein levels via WB. **E** The m^6^A levels of METTL3-knockdown cells detected via dot blot. **F** CCK-8 assays showed the relative cell viability in shNC and shMETTL3 cells with different doses of OXA treatment for 48 h. **G** IC50 of shNC and shMETTL3 cells calculated according to the CCK-8 assays. **H** The colony formation assays performed to detect the survival of Huh7 cells exposed to oxaliplatin with or without METTL3 silencing (left panel). The relative viability curve was shown (middle panel) with IC50 values calculated (right panel). **I** Colony formation assays were conducted with oxaliplatin, STM2457, and their combination at various concentrations (left panel). Combination indices of STM2457 and OXA in Huh7 cells calculated with CalcuSyn software (right panel). **J** Combination indices of STM2457 and OXA in Huh7 and HepG2 cells based on CCK-8 assays calculated with CalcuSyn software indicated a synergistic anti-tumor effect. Data are presented as mean ± SD (*p < 0.05, **p < 0.01, and ***p < 0.001).

Emerging evidence has suggested that aberrant epigenetic regulations contribute to drug resistance of human cancers [16]. To identify the epigenetic vulnerabilities that influence the responses of HCC cells to oxaliplatin, we carried out a siRNA library targeting key regulators of prevalent epigenetic modifications, including 5-methylcytosine (m^5^C), N^6^-methyladenosine (m^6^A), pseudouridine (Ψ), DNA methylation, histone acetylation, and histone methylation. Our results from independent siRNA screening experiments of Huh7, HepG2, and PLC/PRF/5 showed that silencing KDM1A or METTL3 consistently increased OXA sensitivity across all cell lines (Fig. 1A–C). The results were consistent with the previous report that KDM1A has been found to be associated with OXA resistance in HCC [15], confirming the good performance of our siRNA screening. Given that METTL3 knockdown conferred oxaliplatin sensitivity across the three cell lines and was among the top-ranked factors in the screening, we focused on METTL3 in this investigation. To explore the role of METTL3 in the development and progression of HCC, we first compared its expression level between HCC tissues and the adjacent liver tissues with western blotting. Our result showed that METTL3 expression was significantly upregulated in HCC tissues compared to their corresponding normal liver tissues (Fig. S1A). Moreover, the data from the TCGA database revealed a negative correlation between *METTL3* expression level and overall survival of HCC patients (Fig. S1B). Thus, our data indicated that METTL3 may drive oxaliplatin resistance and act as an oncogene during liver tumorigenesis.

To verify the effect of METTL3 on HCC resistance to OXA, we generated the stable METTL3-depleted cells in Huh7 and HepG2 (Fig. 1D). First, METTL3 depletion conferred the reduction of the global RNA m^6^A level as confirmed by m^6^A dot-blot assays (Fig. 1E). Furthermore, knockdown of METTL3 resulted in enhanced sensitivity to oxaliplatin treatment, as predicted by the siRNA screening findings (Fig. 1F). Additionally, the half maximal inhibitory concentration (IC50) values of the METTL3-depleted cells were significantly lower than those of the control cells (Fig. 1G). Silencing METTL3 significantly inhibited HCC cell colony formation after exposure to oxaliplatin (Fig. 1H), but the effects were not as pronounced with other anticancer agents including cisplatin (DDP) and 5-FU (Fig. S1C), indicating that the hypersensitivity to oxaliplatin may indeed be more specific to its mode of action. Considering that METTL3 is the primary catalytic enzyme responsible for m^6^A methylation [17], we utilized STM2457, a targeted inhibitor that specifically blocks the S-adenosylmethionine (SAM) binding site of METTL3, to determine the role of m^6^A catalytic function of METTL3 in OXA resistance. Our data showed that STM2457 inhibited HCC cells (including Huh7 and HepG2) proliferation in a concentration-dependent manner while it did not obviously affect the proliferation of normal liver cell LO2 (Fig. S1D–F). Compared to HCC cells treated with OXA alone, HCC cells treated with the combination of STM2457 and OXA exhibited significantly lower cell viability. Furthermore, the combination indices of STM2457 with OXA were less than 1, indicating a synergistic effect (Fig. 1I, J). Taken together, these data suggested that METTL3-dependent m^6^A modification plays a critical role in driving HCC resistance to oxaliplatin.

### METTL3 depletion inhibits the activity of the pentose phosphate pathway

To uncover the role of METTL3 in oxaliplatin resistance, we performed RNA-seq to analyze gene expression changes after silencing METTL3. Our results identified 1470 differentially expressed genes, including 598 upregulated genes with fold change (FC) > 1.5 and 872 downregulated genes with FC < 0.66. Interestingly, biological process enrichment analysis showed that METTL3 knockdown affected pathways related to metabolism (Fig. S2A, B). Furthermore, GSEA analysis confirmed the connection between METTL3 and metabolism in HCC (Fig. S2C). To further investigate biological changes involved in metabolism, we performed a non-targeted metabolomic analysis on Huh7-shNC and Huh7-shMETTL3 groups. Each group included five independent repeats. The OPLS-DA model and Pearson correlation map showed a marked difference in the metabolic profiles following METTL3 knockdown (Fig. S2D). Among the critical metabolites, we observed a decrease in carbohydrates, nucleotides, and alkylamines, while there was an increase in organic acids and alcohols (Fig. S2E). Enrichment analysis using Metaboanalyst website (https://www.metaboanalyst.ca/) revealed the pentose phosphate pathway as the top-ranked pathway (Fig. S2F), with the top differential metabolite being 6-phosphogluconic acid, a crucial metabolite of the oxidative phase of the pentose phosphate pathway (PPP) (FC = 0.09, *p* = 0.017) (Fig. S2G). Another oxidative PPP-related metabolite, D-Ribulose-5-phosphate, was also significantly reduced in the shMETTL3 group (FC = 0.358, *p* = 0.00045). These data imply that silencing METTL3 may alter the metabolic processes of HCC, resulting in the impairment of the oxidative phase of PPP.

### METTL3 regulates oxaliplatin resistance through G6PD

**Fig. 2:**
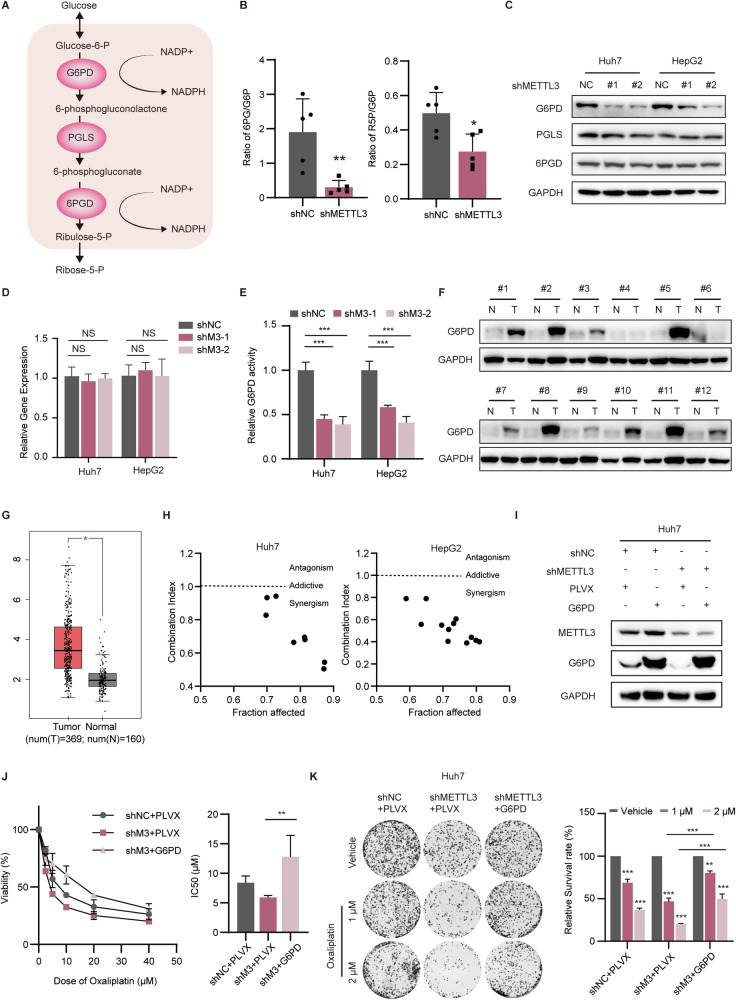
METTL3 regulates OXA susceptibility through G6PD. **A** Diagram of the oxidative phase of the phosphate pentose pathway. **B** Ratio of 6-phosphogluconic acid/Glucose-6-phosphate (6PG/G6P) and D-Ribulose-5-phosphate/Glucose-6-phosphate (R5P/G6P) according to the non-targeted metabolomics. **C** The protein levels of three key enzymes in oxidative PPP detected via WB upon METTL3 silencing. **D** Relative RNA levels of G6PD in Huh7 and HepG2 cells detected via real-time PCR upon METTL3 silencing. **E** Relative G6PD activity determined in HCC cells with METTL3 silencing. **F** Expression of G6PD detected via WB in 12 pairs of frozen HCC tissues. **G** Expression of G6PD in liver cancer and adjacent normal tissues from TCGA database. **H** Combination indices of DHEA and OXA in Huh7 and HepG2 cells calculated with CalcuSyn software indicated a synergistic anti-tumor effect. **I** WB detection of G6PD and METTL3 expression in shMETTL3 and control Huh7 cells with or without G6PD overexpression. **J** CCK-8 assays showed the relative cell viability in shNC and shMETTL3 cells with or without G6PD overexpression following OXA treatment for 48 h (left panel). IC50 of shNC + PLVX, shMETTL3 + PLVX, and shMETTL3 + G6PD in Huh7 cells calculated according to the CCK-8 assays (right panel). **K** The colony formation assays performed to detect the survival of shNC + PLVX, shMETTL3 + PLVX, and shMETTL3 + G6PD cells following different doses of OXA treatment (left panel). The relative survival rate calculated was shown (right panel). Data are presented as mean ± SD (*p < 0.05, **p < 0.01, and ***p < 0.001).

In order to investigate the role of METTL3 in the oxidative phase of PPP, we conducted an analysis of the enzymes involved in the pathway (Fig. 2A): glucose-6-phosphate 1-dehydrogenase (G6PD), 6-phosphogluconolactonase (PGLS), and 6-phosphogluconate dehydrogenase (6PGD) [18]. Our metabolomics data revealed a significant decrease in the ratio of ribulose-5-P (R5P) and 6-phosphogluconate (6PG) to glucose-6-phosphate (G6P) after silencing METTL3, while 6-phosphogluconolactone was not detectable (Fig. 2B). These findings suggest that the activity of either glucose-6-phosphate 1-dehydrogenase (G6PD) or 6-phosphogluconolactonase (PGLS) may be impaired. Further Western blotting assays showed a reduction in G6PD expression after METTL3 silencing, while the mRNA level of G6PD remained unchanged (Fig. 2C, D). As expected, silencing METTL3 led to a reduction in G6PD activity (Fig. 2E). G6PD has been found to play an oncogenic role in various cancers [19]. Consistently, further analysis of HCC samples from the TCGA database and SYSUCC revealed that G6PD expression was significantly upregulated in HCC tissues and was positively correlated with poor overall survival (Figs. 2F, G, S3A). We then tested the effect of dehydroepiandrosterone (DHEA), a specific inhibitor of G6PD, on HCC cells and found that it enhanced their sensitivity to OXA (Fig. 2H). To confirm the role of G6PD in the METTL3-mediated OXA susceptibility of HCC cells, we performed ectopic expression of G6PD in METTL3-silenced cells and observed a rescue of METTL3-silenced induced OXA susceptibility in both CCK-8 and colony formation assays (Fig. 2I–K). These results indicate that G6PD partially mediates METTL3-induced sensitivity changes in HCC cells to OXA.

### Silencing METTL3 impairs G6PD protein stability through E3 ligase TRIM21

**Fig. 3:**
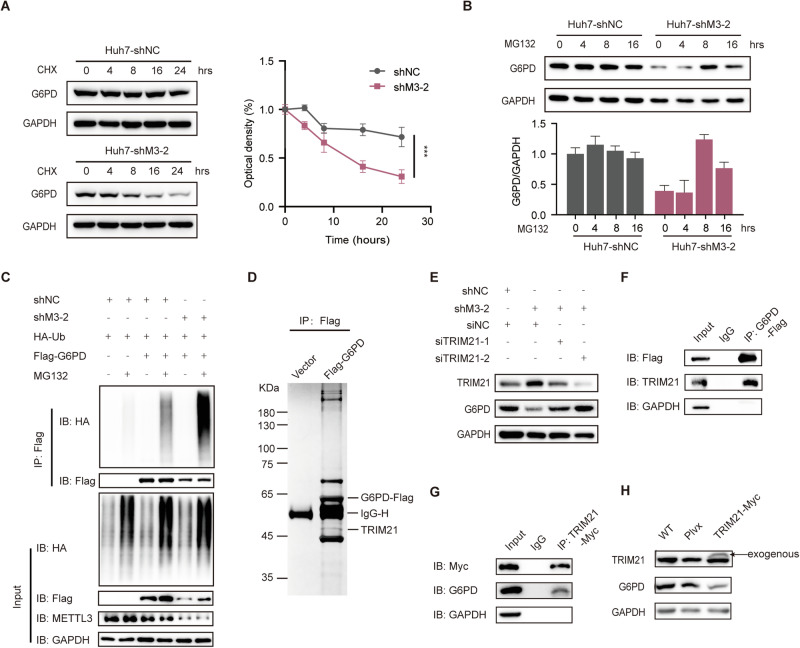
METTL3 regulates protein stability of G6PD via E3 ligase TRIM21. **A** METTL3-silencing and control cells were treated with CHX for the indicated time, and the protein levels of G6PD and GAPDH were determined by WB (left panel). The quantitation data are shown in the right panel. **B** METTL3-silencing and control cells were treated with MG132 for the indicated time, and the protein levels of G6PD and GAPDH were determined by WB. **C** METTL3-silencing and control cells were transfected with indicated plasmids and then incubated with MG132. The levels of different proteins were detected with WB. **D** The lysate extracted from G6PD-Flag-overexpressing or control Huh7 cells was pulled down by anti-Flag beads and resolved by SDS-PAGE. The silver-stained gel showed differential bands with G6PD and TRIM21 highlighted. **E** Protein levels of TRIM21, G6PD, and GAPDH were determined in shMETTL3 Huh7 cells with or without siTRIM21 transfection. **F** Huh7 cells transfected with G6PD-Flag were subjected to immunoprecipitation using anti-Flag antibody or IgG control. The protein expression was detected via WB. **G** Huh7 cells transfected with TRIM21-Myc were subjected to immunoprecipitation using anti-Myc antibody or IgG control. The protein expression was detected via WB. **H** Protein levels of TRIM21, G6PD, and GAPDH were determined in TRIM21-Myc overexpression and control cells. Data are presented as mean ± SD (*p < 0.05, **p < 0.01, and ***p < 0.001).

To elucidate the mechanism by which METTL3 regulates G6PD expression, we conducted an examination of G6PD at the transcriptional level. Surprisingly, our RT-PCR results revealed no significant alteration in the mRNA level of G6PD (Fig. 2D). To delve deeper into the post-transcriptional regulation of G6PD, we evaluated protein stability by treating control and shMETTL3 cells with cycloheximide (CHX), a protein synthesis inhibitor. Our immunoblot analysis unveiled a notable decrease in the half-life of G6PD protein following METTL3 knockdown (Fig. 3A). Moreover, we explored the effects of inhibiting the proteasome and lysosome on cellular G6PD levels in METTL3-knocked-down cells. Remarkably, treatment with the proteasome inhibitor MG132, but not the lysosome inhibitors, significantly increased cellular G6PD levels (Figs. 3B, S3B). To further investigate whether METTL3 influences G6PD stability through the ubiquitin-proteasome pathway, we transfected ubiquitin-HA and G6PD-Flag vectors into Huh7 stable transfectants with or without MG132 treatment. The results of our immunoblot analysis demonstrated that shMETTL3 substantially enhanced the ubiquitination level of G6PD (Fig. 3C). These findings strongly suggest that METTL3 may impact G6PD stability through the ubiquitin-proteasome pathway.

To identify the E3 ligase responsible for G6PD degradation, we performed co-immunoprecipitation followed by mass spectrometric detection in Huh7 cells expressing G6PD-Flag (Figs. 3D, S3C). We then constructed a siRNA library consisting of potential E3 ligases obtained from MS analysis, literature, and the UbiBrowser database (http://ubibrowser.bio-it.cn) [20, 21]. Specific siRNA screening revealed that only TRIM21, a protein identified in the MS analysis and reported in the literature [21], was able to rescue the expression of G6PD in shMETTL3 cells (Figs. 3E, S3D). Furthermore, subsequent immunoblot assays confirmed the interaction between G6PD and TRIM21 (Fig. 3F), which was supported by the reciprocal co-immunoprecipitation (Fig. 3G). Notably, overexpression of TRIM21 resulted in a reduction in G6PD expression (Fig. 3H). Collectively, these findings indicate that METTL3 regulates G6PD expression through TRIM21-mediated protein stability alteration.

### YTHDF2 regulates TRIM21 mRNA stability through the m^6^A-methylated 3′UTR

**Fig. 4:**
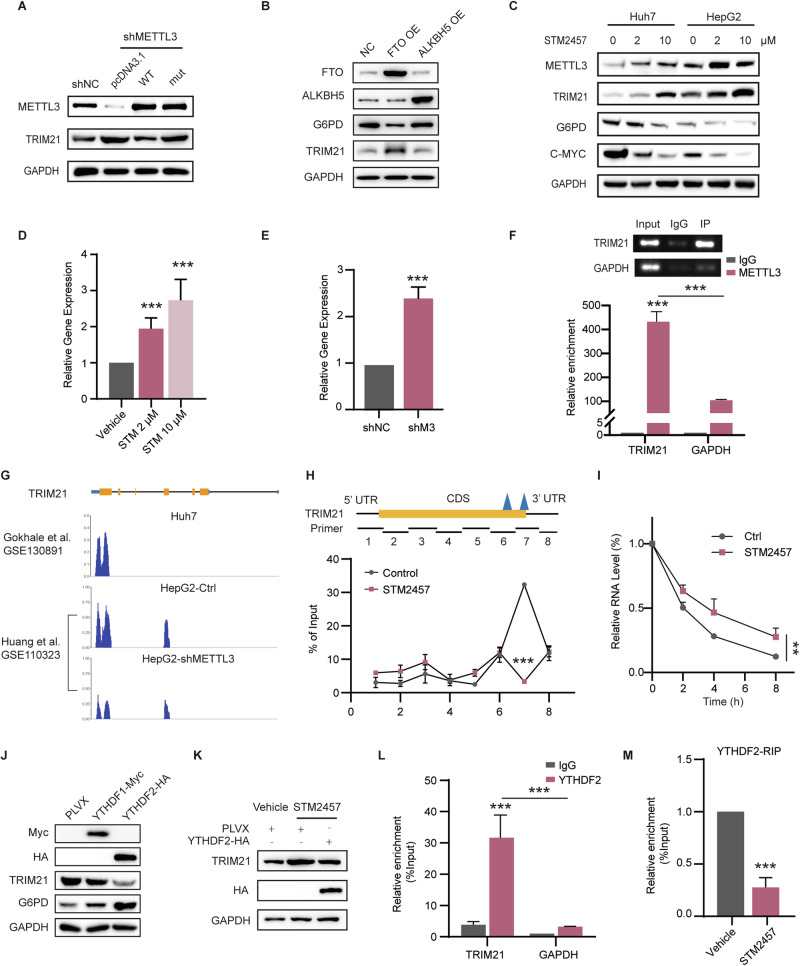
METTL3 affects mRNA stability of TRIM21 via m^6^A-YTHDF2. **A** The expression of TRIM21, METTL3, and GAPDH was determined in Huh7-shNC, shMETTL3 transfected with METTL3-WT, METTL3-mut, or control plasmids. **B** The expression of TRIM21, G6PD, and GAPDH was determined in Huh7 cells transfected with FTO, ALKBH5, or control plasmids. **C** Changes of indicated proteins with different doses of STM2457 treatment detected via WB. **D** Results of qPCR showed the effect of different doses of STM2457 treatment on the expression of TRIM21 in Huh7 cells. GAPDH was used as the control. **E** Results of qPCR showed expression of TRIM21 in Huh7-shMETTL3 or control cells. GAPDH was used as the control. **F** Agarose electrophoresis and real-time PCR showing the abundance of TRIM21 and GAPDH in Huh7 cells immunoprecipitated with anti-METTL3 antibody or IgG control. **G** m^6^A modifications of TRIM21 mRNA in Huh7 and HepG2 cells from RMVar database. shMETTL3 in HepG2 diminished m^6^A modifications of TRIM21. **H** MeRIP was performed in Huh7 cells treated with STM2457 or vehicle using an anti-m^6^A antibody. qPCR showed the m^6^A modification levels of different fractions of TRIM21 detected with eight pairs of primers overlapping the whole length of TRIM21 mRNA. The diagram of the primers is shown above, with the blue triangle indicating the m^6^A-modification enriched sites. **I** qPCR analysis of TRIM21 decay rates in Huh7 cells treated with STM2457 or vehicle was performed at specified time points following Act-D treatment. The expression of TRIM21 was normalized to U6. **J** WB determined the expression of TRIM21, G6PD, and GAPDH in Huh7 cells transfected with YTHDF1-Myc, YTHDF2-HA, or control plasmids. **K** WB determined the expression of TRIM21 in Huh7 cells incubated with STM2457 or vehicle and transfected with YTHDF2 or control vectors. **L** Real-time PCR showing the abundance of TRIM21 and GAPDH in Huh7 cells immunoprecipitated with anti-YTHDF2 antibody or IgG control. **M** Real-time PCR showing the abundance of TRIM21 in Huh7 cells immunoprecipitated with anti-YTHDF2 antibody following STM2457 or vehicle treatment. Data are presented as mean ± SD (*p < 0.05, **p < 0.01, and ***p < 0.001).

We further investigated the mechanism by which METTL3 regulated TRIM21 expression. Given recent reports on the non-m^6^A functions of METTL3 in tumorigenesis [22], we aimed to determine whether METTL3 regulates TRIM21 through an m^6^A-dependent manner. To test this, we conducted rescue experiments in stable METTL3-silenced cells using wild-type METTL3 and a catalytic mutant METTL3 (aa395-398, DPPW-APPA). Interestingly, only the wild-type METTL3 was able to rescue the elevated expression of TRIM21 resulting from METTL3 knockdown (Fig. 4A). To explore the role of m^6^A demethylases in G6PD regulation, we ectopically expressed the m^6^A demethylase FTO in Huh7 cells. Notably, the expression of G6PD was reduced upon FTO overexpression, whereas ALKBH5 had no significant effect (Fig. 4B). Additionally, treatment with the METTL3-specific catalytic inhibitor, STM2457, exhibited dose-dependent upregulation of TRIM21 expression and downregulation of G6PD expression. As a positive control, the expression of C-MYC, known to be regulated by METTL3 through m^6^A methylation, was also modulated by STM2457 treatment (Fig. 4C). Interestingly, the expression of METTL3 was slightly increased, suggesting the presence of feedback regulatory mechanisms to maintain METTL3’s methylation function. Moreover, the mRNA level of TRIM21 was significantly enhanced following STM2457 treatment or shMETTL3 (Fig. 4D, E). Despite the known role of C-MYC in OXA resistance, it failed to significantly reverse METTL3-silencing-induced oxaliplatin sensitization in our study (Fig. S3E–G). These findings collectively support the notion that METTL3 regulates TRIM21 through an m^6^A-dependent manner.

Furthermore, we conducted RNA immunoprecipitation followed by quantitative PCR (RIP-qPCR) to examine the direct interaction between METTL3 and TRIM21 mRNA. The results revealed specific binding of METTL3 to TRIM21 mRNA, with GAPDH serving as a negative control (Fig. 4F). To determine the precise m^6^A site regulated by METTL3, we analyzed the data from the RMVar database (https://rmvar.renlab.org/) [23]. Interestingly, we identified two distinct peaks near the stop codon of the 3′UTR of TRIM21 in both HCC cells and common model cells. Notably, these peaks were significantly diminished upon METTL3 silencing or mutation of the catalytic domain (Figs. 4G, S4A). To narrow down the specific enriched domain, we designed eight primer sets covering the entire length of TRIM21 mRNA. Through m^6^A-specific immunoprecipitation followed by qPCR, we found that the domain spanning from 1333 to 1637 bp (primer 7) of TRIM21 mRNA exhibited a significant enrichment with the anti-m^6^A antibody. Importantly, this enrichment was reduced when treated with STM2457, confirming the presence of m^6^A modification at this specific region (Fig. 4H).

To gain insight into how METTL3 regulates TRIM21 through m^6^A modification, we conducted experiments to investigate the stability of TRIM21 at both the RNA and protein levels. We used actinomycin D (Act-D) to block transcription and CHX to inhibit protein synthesis. Surprisingly, we observed that in cells treated with the METTL3-specific inhibitor STM2457, the half-life of TRIM21 mRNA was significantly prolonged compared to control cells, suggesting that m^6^A methylation, facilitated by METTL3, attenuated the stability of TRIM21 mRNA (Fig. 4I). However, the protein stability of TRIM21 remained unaffected under these conditions (Fig. S4B). Among the known m^6^A readers, YTHDF2 has been implicated in modulating the stability of target mRNAs [24]. To identify the specific m^6^A reader involved in TRIM21 regulation, we overexpressed YTHDF1 and YTHDF2 in Huh7 cells. Interestingly, only YTHDF2 was able to down-regulate the expression of TRIM21 (Fig. 4J). Furthermore, we found that forced expression of YTHDF2 rescued the elevated TRIM21 expression caused by STM2457 treatment (Fig. 4K). Subsequent RNA immunoprecipitation followed by qPCR (RIP-qPCR) assays demonstrated the direct interaction between YTHDF2 and TRIM21 mRNA, confirming its role as an m^6^A reader for TRIM21 (Fig. 4L). Importantly, this interaction was weakened upon STM2457 treatment (Fig. 4M), supporting the involvement of m^6^A modification in the regulation of TRIM21. Taken together, our findings highlight the crucial role of YTHDF2 as an m^6^A reader in governing the stability of TRIM21 mRNA by recognizing the m^6^A-modified site within the 3’UTR of TRIM21.

### Silencing METTL3 disrupts the cell cycle by reducing nucleotide synthesis and increasing oxidative damage

**Fig. 5:**
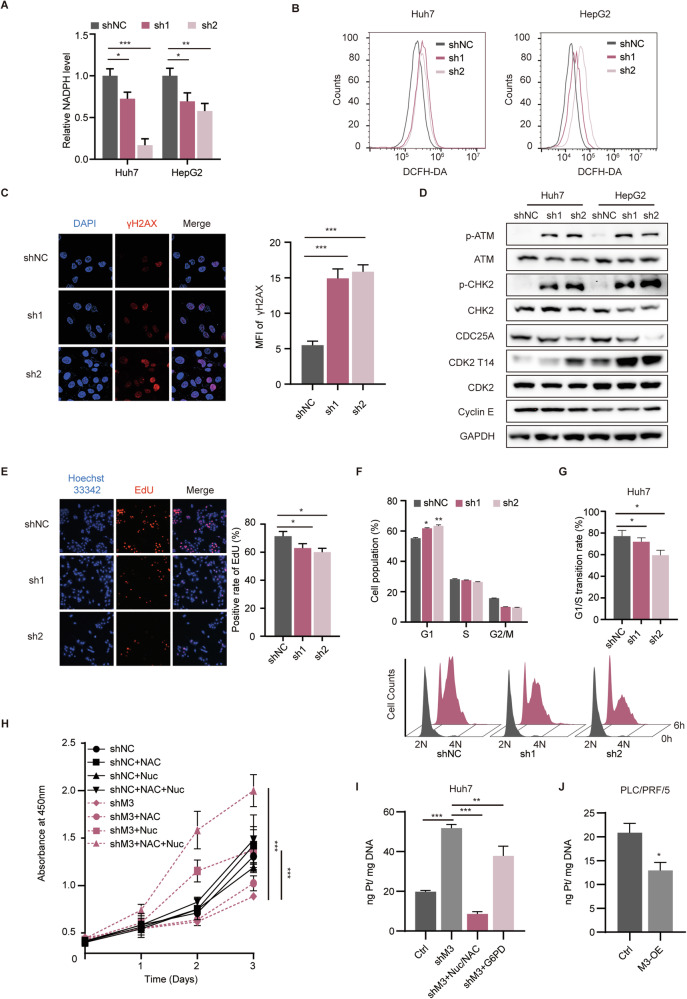
Silencing METTL3 blocked cell cycle due to reduced nucleotide synthesis and increased oxidative damage. **A** Relative NADPH levels were determined in HCC cells with METTL3 silencing. **B** ROS levels of HCC cells with METTL3 silencing were determined via FCS using a DCFH-DA probe. **C** The level of γH2AX in METTL3-silencing Huh7 cells was determined via IF (left panel), and the MFI of γH2AX was counted (right panel). **D** Detection of proteins in ATM-CHK2 DNA damage response pathway and proteins responsible for G1/S transition in METTL3 silencing and control cells. **E** EdU assay images (left panel) were obtained from METTL3-silencing and control Huh7 cells with the quantification of positive cells shown in the right panel. **F** Cell cycle distributions of METTL3-silencing Huh7 cells were determined via FCS following PI and RNase treatment. **G** Cell cycle release assays showing the transition of G1/S with thymidine used for synchronization (lower panel). Statistics of G1/S transition rates were shown in the upper panel. **H** CCK-8 proliferation assays showing the rescue effect of NAC and deoxyribonucleosides (Nuc) in METTL3-silencing or control Huh7 cells. **I** The DNA platination detected via ICP-MS in Huh7 cells with different treatments. **J** The DNA platination detected via ICP-MS in both PLC/PRF/5 control cells and METTL3-overexpression cells. Data are presented as mean ± SD (*p < 0.05, **p < 0.01, and ***p < 0.001).

PPP consists of two key phases: the oxidative phase and the non-oxidative phase. The oxidative phase is essential for generating R5P for nucleotide biosynthesis and NADPH, which is critical for maintaining the cellular redox balance. Consequently, the PPP is vital in supporting the anabolic needs and mitigating oxidative stress in cancer cells [25]. In our study, we observed a blockade of the PPP pathway following METTL3 knockdown, which led us to investigate its implications on the redox state and DNA synthesis in HCC cells. As anticipated, the knockdown of METTL3 resulted in a reduction in NADPH level (Fig. 5A) and a notable increase in ROS (Fig. 5B). Moreover, the METTL3-knockdown cells exhibited heightened susceptibility to apoptosis when exposed to H_2_O_2_, indicating a compromised defense mechanism against oxidative stress (Fig. S5A). Importantly, excessive ROS generation is known to induce DNA double-strand breaks [26]. Consistent with this, our immunofluorescence assays showed a pronounced increase in γH2AX, a marker of DNA damage, following METTL3 silencing (Fig. 5C). Complementing these findings, the comet assay demonstrated increased DNA damage in METTL3-silenced cells, as evidenced by the elongated comet tails (Fig. S5B). Furthermore, immunoblot analysis revealed elevated phosphorylation levels of ATM and CHK2, indicating activation of the DNA damage response pathway, while the total protein levels remained unchanged (Fig. 5D).

In addition to its role in redox state maintenance, PPP is crucial for providing nucleic acid precursors. To evaluate the impact of METTL3 on DNA synthesis, we performed EdU incorporation assays. Remarkably, DNA synthesis was significantly diminished following METTL3 knockdown (Fig. 5E). Both impaired DNA synthesis and DNA damage can impede the cell cycle. Analysis of the cell cycle distribution revealed G0/G1 arrest in cells with METTL3 silencing (Fig. 5F). Subsequent cell cycle release assays confirmed the G1/S transition impairment (Figs. 5G, S5D). Previous studies have suggested that activation of the ATM/CHK2 axis can trigger G0/G1 cell cycle arrest via the CDC25A/CDK2 signaling pathway [27]. WB analysis showed a decrease in CDC25A protein levels along with increased phosphorylation at the inhibitory site T14 of CDK2. Notably, there was no change in the expression of cyclin E, which is responsible for G1/S transition (Fig. 5D). To investigate whether PPP repression-induced cell cycle arrest plays a prominent role in growth inhibition, we cultured control and shMETTL3 cells in a medium supplemented with an excess of the ROS scavenger NAC and/or nucleosides (dNTPs). It was observed that either NAC or nucleotides alone could partially rescue the growth of shMETTL3 cells, although to a lesser extent compared to the combined usage of both (Fig. 5H). Conversely, neither Ferr-1 (the inhibitor of ferroptosis) nor Z-VAD-FMK (the inhibitor of apoptosis) could rescue the proliferation inhibition induced by knocking down METTL3 (Fig. S5F). Interestingly, the combination treatment had a more pronounced effect on METTL3-silencing cells compared to the controls, likely due to differences in exogenous nucleotide utilization. Further investigation revealed that the expression of Concentrative Nucleoside Transporters (CNTs), which are responsible for nucleoside uptake, increased following METTL3 silencing (Fig. S5E). Additionally, METTL3 overexpression significantly reduced oxaliplatin-induced DNA platination (Fig. 5I). The increase in DNA platination observed in METTL3-knockdown cells could be partially mitigated by G6PD activation or supplementation with nucleotides and NAC, both products of the PPP (Fig. 5J). Furthermore, oxaliplatin-induced DNA repair activation was reduced following METTL3 activation, as evidenced by the phosphorylation status of H2AX, ATM/ATR, and their downstream targets CHK1/CHK2 (Fig. S5C).

Next, we investigated the impact of METTL3 on tumor proliferation in vitro and in vivo. In vitro studies using colony formation and CCK-8 assays demonstrated that METTL3 knockdown inhibited cell proliferation, while overexpression of METTL3 through transfection with a pcDNA3.1-METTL3 plasmid promoted tumor proliferation (Fig. S6A, B). To validate these findings in an in vivo setting, we performed a subcutaneously transplanted tumor model experiment. Our results demonstrated that silencing METTL3 significantly impeded tumor growth, leading to a notable reduction in tumor volumes and weights (Fig. S6C). Furthermore, immunostaining for the proliferation marker Ki-67 confirmed decreased expression in the shMETTL3 group compared to the control (Fig. S6D). Considering the known role of G6PD in promoting HCC progression, we explored its involvement in our observations. Remarkably, silencing G6PD resulted in reduced cell proliferation (Fig. S6E, F), while ectopic expression of G6PD could partially rescue the cell proliferation repression induced by METTL3 knockdown (Fig. S6G, H). Taken together, our findings provide compelling evidence that silencing METTL3 in HCC cells leads to elevated ROS levels and impaired nucleotide synthesis, ultimately resulting in cell cycle arrest and inhibition of HCC growth.

### Silencing METTL3 sensitizes HCC cells to oxaliplatin treatment in vivo

**Fig. 6:**
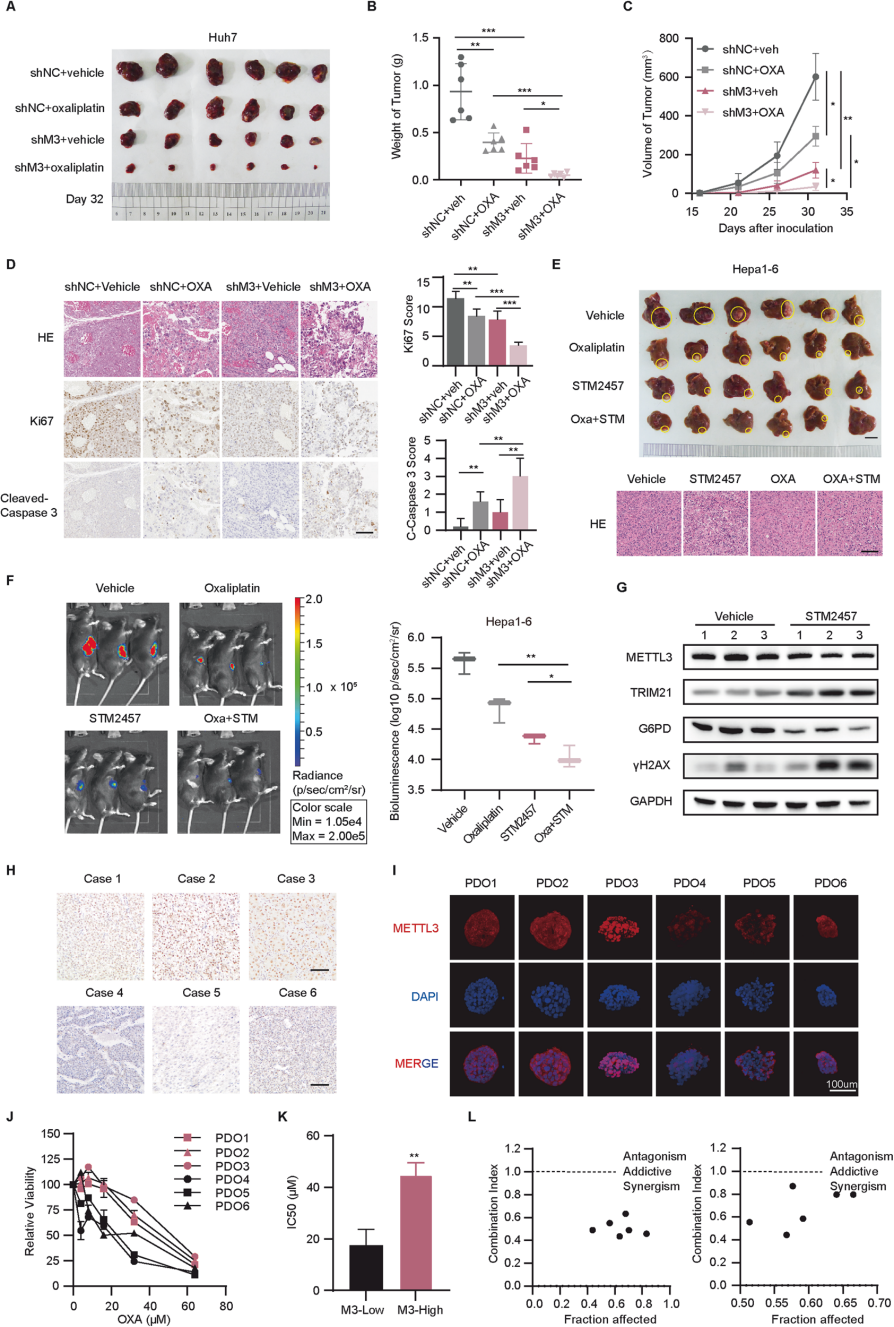
Silencing METTL3 sensitizes HCC cells to oxaliplatin treatment in vivo and in organoid models. The image (**A**) and the weights (**B**) of subcutaneous xenografts from NOD/SCID mice. Mice received OXA or vehicle ten days after Huh7-shNC or shMETTL3 cells plantation and were sacrificed on Day 32. **C** Tumor growth of subcutaneous xenografts in NOD/SCID mice was monitored every 5 days by measuring the length (L) and width (W) with a caliper. And the tumor volume (V) was calculated according to the formula V = (L × W^2^)/2. **D** IHC images of Ki-67 and c-caspase and HE images detected in subcutaneous xenografts from NOD/SCID mice (left panel). The statistics of expression of Ki-67 and c-caspase were shown in the right panel. Scale bar: 100 μm. **E** General images (upper) and HE images (lower) of orthotopic xenografts from C57BL/6 J mice. The mice received indicated medicine five days after laparotomy and were sacrificed on Day 24. Scale bar: 100 μm. **F** The in vivo imaging of orthotopic C57BL/6 J mice with an injection of D-luciferin i.p. (left panel). The statistics of the bioluminescence log values for each group are shown in the right panel. **G** WB detection of indicated protein in orthotopic tumors of C57BL/6 J mice following treatment of vehicle or STM2457. Each group contained three animals. **H** Representative IHC staining images of METTL3 in HCC specimens. Scale bar: 100 μm. **I** Representative IF staining images of METTL3 in HCC organoids. Scale bar: 100 μm. **J** Dose-response curves for OXA for 5 days in HCC organoids. **K** Average IC50 values of HCC organoids stratified by METTL3 expression level. **L** Combination indices of STM2457 and OXA on HCC organoids calculated with CalcuSyn software. Data are presented as mean ± SD (*p < 0.05, **p < 0.01, and ***p < 0.001).

To investigate the potential therapeutic implications of targeting METTL3 in HCC, we conducted in vivo experiments to assess the effect of METTL3 knockdown and its interaction with oxaliplatin treatment. Subcutaneous injection of METTL3 knockdown or control Huh7 cells was performed in mice, followed by the administration of OXA every 3 days for a duration of 3 weeks. Consistent with our in vitro findings, the results demonstrated that METTL3 silencing significantly enhanced the sensitivity of HCC cells to OXA in the in vivo setting (Fig. 6A–C). Immunohistochemical analysis further revealed that the combination of METTL3 silencing and OXA treatment resulted in decreased proliferation, as evidenced by reduced Ki-67 staining, and increased apoptosis, as indicated by elevated levels of cleaved caspase 3 (Fig. 6D).

To further explore the clinical applicability of targeting METTL3 in HCC therapy, we investigated the impact of the METTL3 inhibitor, STM2457, on the susceptibility of HCC to OXA in orthotopic tumor models. Treatment with either STM2457 or OXA alone resulted in significant impairment of tumor growth without affecting body weights or causing discernible toxicity (Figs. 6E, F, S7A, B). Importantly, histological examination of normal tissues revealed no significant alterations (Fig. S7C). Furthermore, immunoblot analysis of orthotopic tumors confirmed the upregulation of TRIM21 and downregulation of G6PD following STM2457 treatment (Fig. 6G). To further explore the impact of STM2457 on OXA sensitivity, we constructed HCC organoids (PDOs) from fresh tissue samples. Double immunofluorescence staining with HepPar-1 and Arg1 confirmed the origin of the PDOs (Fig. S7D). Based on METTL3 expression levels, six PDOs were categorized into high-expression and low-expression groups (Fig. 6H, I). PDOs in the high-expression group showed greater viability under OXA treatment compared to the low-expression group (Figs. 6J, S7E). The IC50 value analysis revealed that the average IC50 value of the high-expression group was significantly higher than that of the low-expression group, indicating a negative correlation between METTL3 expression and OXA sensitivity (Fig. 6K). Furthermore, the combined application of STM2457 and OXA demonstrated a synergistic effect on HCC PDOs, as evidenced by the CI values (Fig. 6L). Our findings underscore the promising therapeutic implications of targeting METTL3 in HCC therapy and provide a rationale for further exploration in clinical settings.

### The correlation between METTL3, G6PD, and treatment responses to OXA-based HAIC in HCC

In light of the previously identified mechanism, we conducted a clinical investigation to explore the association between the expression levels of METTL3 and G6PD, as determined by immunochemistry (IHC) assays, and the responses to OXA-based HAIC in HCC. We collected biopsy samples from 49 HCC cases diagnosed with advanced HCC at Sun Yat-sen University Cancer Center between May 9, 2017, and December 19, 2017, before initiating OXA-based HAIC therapy. High expression of METTL3 or G6PD was defined as having an H score higher than the median score, while low expression was categorized as equal to or lower than the median. Treatment responses were assessed using Response Evaluation Criteria in Solid Tumors (RECIST; version 1.1) and modified RECIST (mRECIST).

**Fig. 7:**
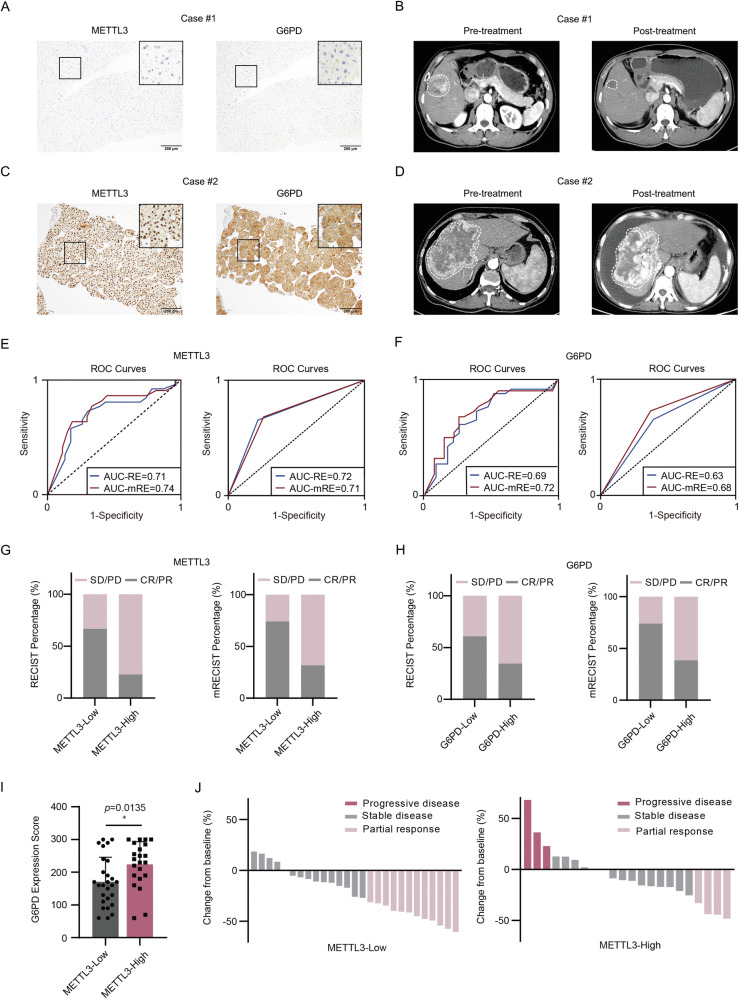
METTL3 expression correlates with G6PD expression and treatment responses to OXA-based HAIC in HCC. **A** Representative images showing both low expression of METTL3 and G6PD in a biopsy of an HCC case. **B** Pre-treatment and post-treatment CT scan images of HCC in a patient with low expression of METTL3 and G6PD. **C** Representative images showing both high expression of METTL3 and G6PD in a biopsy of an HCC case. **D** Pre-treatment and post-treatment CT scan images of HCC in a patient with high expression of METTL3 and G6PD. **E** ROC curve analyses of METTL3 expression (left panel: continuous score; right panel: binary category) for predicting the treatment responses to OXA-based HAIC using the RECIST and modified RECIST criteria. **F** ROC curve analyses of G6PD expression (left panel: continuous score; right panel: binary category) for predicting the treatment responses to OXA-based HAIC using the RECIST and modified RECIST criteria. **G** Correlation between therapeutic efficacy and METTL3 expression as assessed by RESIST and mRESIST criteria. **H** Correlation between therapeutic efficacy and G6PD expression as assessed by RESIST and mRESIST criteria. PR Partial Response, CR Complete Response, SD Stable Disease, PD Progressive Disease. **I** The correlation between the expression of METTL3 and G6PD in a HAIC-treated HCC cohort. **J** Waterfall plots depicting the maximum response of intrahepatic target lesions treated with OXA-based HAIC in the METTL3-low and -high groups.

Our findings demonstrated that patients with low expression of METTL3 or G6PD displayed a favorable response to HAIC treatment, as determined by CT scan (Fig. 7A, B). Conversely, high expression of METTL3 or G6PD was associated with resistance to HAIC therapy (Fig. 7C, D). To assess the potential of METTL3 and G6PD as predictive markers for HCC patient response to HAIC treatment, receiver operating characteristic (ROC) curves were constructed, and the area under the curve (AUC) was calculated. METTL3 exhibited AUCs of 0.71 and 0.74 for RECIST and mRECIST criteria, respectively (Fig. 7E, left panel). When converted into binary classification, the AUCs were 0.72 and 0.71 for RECIST and mRECIST criteria, respectively (Fig. 7E, right panel). Similarly, G6PD IHC staining yielded AUCs ranging from 0.63 to 0.72 (Fig. 7F).

In accordance with RECIST and mRECIST criteria, METTL3-high patients showed lower numbers of cases experiencing complete response (CR) or partial response (PR) compared to METTL3-low patients (Fig. 7G). This observation was also evident in the comparison between G6PD-high and G6PD-low groups (Fig. 7H). Importantly, we observed a positive correlation between the expression levels of G6PD and METTL3 in HCC tissues (Fig. 7I). Additionally, patients in the METTL3-high group exhibited higher rates of progressive disease (PD) compared to the METTL3-low group based on RECIST and mRECIST criteria (Fig. 7J). These findings highlight the potential of METTL3 expression as a novel biomarker for predicting the responses to HAIC treatment, indicating that higher METTL3 expression may be associated with poor outcomes in OXA-based therapy.

## Discussion

Immunotherapy has demonstrated significant promise in the treatment of advanced HCC. However, it remains effective in only a subset of patients, underscoring the need for additional therapeutic strategies [28]. As the current standard of care, chemotherapy is extensively employed in managing HCC [29, 30], with oxaliplatin-based HAIC emerging as a particularly effective maintenance therapy for advanced HCC [4, 5]. Postoperative adjuvant HAIC with oxaliplatin has also been shown to significantly improve the overall survival of HCC patients [3]. Despite these advancements, the development of resistance to oxaliplatin poses a substantial challenge to the long-term success of HAIC. Identifying reliable biomarkers to predict patient response and optimize treatment regimens is crucial for improving therapeutic outcomes. Our study identified the METTL3-TRIM21-G6PD axis as a critical regulator of OXA resistance in HCC. Specifically, we demonstrated that METTL3 expression could serve as a predictive biomarker for responses to HAIC treatment. More importantly, the use of the METTL3 inhibitor STM2457 was shown to significantly enhance the therapeutic efficacy of oxaliplatin, offering a potential avenue to overcome chemoresistance.

The mechanisms underlying oxaliplatin resistance are multifactorial, involving processes such as cellular influx/efflux of drugs, DNA adduct repair, regulation of cell death, and epigenetic modifications. Epigenetic mechanisms, as master regulators, play a direct role in cancer chemoresistance by altering the expression of genes involved in DNA damage response, cell-cycle control, apoptosis, and DNA repair pathways [31]. While the role of epigenetics in chemotherapy resistance is increasingly recognized, the specific epigenetic mechanisms driving oxaliplatin resistance remain insufficiently explored. Recent studies have implicated the histone deacetylase LSD1 and the arginine methyltransferase PRMT3 as key drivers of oxaliplatin resistance in HCC [2, 15], highlighting the importance of epigenetic regulation in this context.

To further investigate the epigenetic basis of oxaliplatin resistance, we conducted a siRNA screen targeting writers and erasers of key epigenetic modifications, including m^5^C, m^6^A, Ψ, DNA methylation, histone acetylation, and histone methylation. Our screen identified the m^6^A methyltransferase METTL3 as a crucial determinant of oxaliplatin resistance in HCC. This finding is consistent with the broader role of METTL3 in HCC pathogenesis and progression, where it has been shown to influence processes such as cell proliferation, epithelial-mesenchymal transition, and stemness [32, 33]. Additionally, METTL3 has been implicated in resistance to various therapies across different cancer types. For example, METTL3-mediated stabilization of USP13 RNA has been shown to promote autophagy and imatinib resistance in gastrointestinal stromal tumors [34], while in HCC, METTL3 has been reported to regulate cancer stem cell properties and contribute to lenvatinib resistance via FZD10 [35]. However, our data suggest that the mechanism by which METTL3 confers oxaliplatin resistance may differ from its previously characterized roles, as evidenced by the limited effect of C-MYC overexpression in reversing METTL3 silencing-induced sensitization to oxaliplatin [36–38].

To elucidate the mechanisms underlying METTL3-mediated oxaliplatin resistance, we employed a bottom-up approach that integrated RNA-seq with untargeted metabolomics analysis. This comprehensive analysis revealed significant perturbations in the PPP, a key metabolic pathway linked to drug resistance. The inhibition of cell proliferation observed upon METTL3 knockdown could be reversed by supplementation with N-acetylcysteine and nucleosides, implicating redox homeostasis and ribonucleotide synthesis in the observed resistance. Further investigation revealed that METTL3 regulates the stability of G6PD, the rate-limiting enzyme of the oxidative PPP, by targeting its m^6^A modification for recognition by YTHDF2, leading to its degradation via the E3 ligase TRIM21.

The PPP plays a crucial role in maintaining cellular antioxidant defenses by providing NADPH, which is essential for redox homeostasis and biosynthesis. Increased PPP activity has been associated with drug resistance in various cancers, including oxaliplatin resistance [25]. For instance, POU2F1 has been shown to enhance proliferation, aerobic glycolysis, and PPP activity, contributing to oxaliplatin resistance in CRC [39]. Similarly, disrupting G6PD-mediated NADPH homeostasis has been found to increase oxaliplatin-induced apoptosis in CRC [40]. While targeting G6PD presents a potential strategy to suppress PPP activity and enhance OXA sensitivity, the clinical application of G6PD inhibitors has been limited by toxicity and other challenges [19]. Our study reveals a novel regulatory mechanism of G6PD mediated by METTL3, suggesting alternative therapeutic strategies to circumvent these limitations.

STM2457, a potent, selective, and orally active METTL3 catalytic inhibitor, has shown promising anti-tumor effects in various cancers, including acute myeloid leukemia (AML), intrahepatic cholangiocarcinoma (ICC), and neuroblastoma [41–43]. Our findings indicate that STM2457 can effectively suppress tumor growth and enhance oxaliplatin sensitivity in HCC without significant toxicity. Notably, overexpression of G6PD in METTL3-silenced cells did not fully reverse the sensitizing effect of METTL3 knockdown on OXA, suggesting that additional METTL3 substrates may also contribute to OXA resistance.

In conclusion, our study provides compelling evidence, both in vitro and in vivo, for the crucial role of METTL3, a core component of the m^6^A methyltransferase complex, in mediating OXA resistance in HCC. Mechanistically, METTL3 silencing destabilizes G6PD by upregulating its E3 ligase TRIM21 through m^6^A modification on the 3′UTR, recognized by YTHDF2. Importantly, both silencing METTL3 and STM2457 treatment increase the sensitivity of HCC cells to OXA. Moreover, METTL3 expression may serve as a novel biomarker for predicting responses to HAIC treatment. These results underscore the functional importance of the METTL3/TRIM21/G6PD axis in driving oxaliplatin resistance and present a promising strategy to overcome chemoresistance in HCC.

## Materials and methods

The detailed materials and methods are provided in the Supporting Information.

## Supplementary information

Supplementary materials

Supplementary tables

Original images of WB

## Data Availability

The LC-MS/MS data for interactive proteomics of G6PD have been deposited to the PRIDE repositories with the accession PXD044817. The RNA-seq data of Huh7 with or without METTL3-silencing have been deposited to the GEO database with the accession GSE241802.
