# Supplementary material for: METTL3 confers oxaliplatin resistance through the activation of G6PD-enhanced pentose phosphate pathway in hepatocellular carcinoma
Source: Cell Death Differ. 2024 Oct 29;32(3):466–79. doi: 10.1038/s41418-024-01406-2 (PMC11894169; doi:10.1038/s41418-024-01406-2)
Supplement: Supplementary file 1 — Supplementary materials [file 41418_2024_1406_MOESM1_ESM.docx]

**Supplementary materials and methods**

**Cell culture.** The liver cancer cell line Huh7 was obtained from National Collection of Authenticated Cell Cultures. HepG2 and PLC/PRF/5 were obtained from ATCC. These cells were cultured in DMEM medium (GIBCO, USA) supplemented with 10% fetal bovine serum (FBS, GIBCO). The cells were maintained in a humidified incubator at 37℃ with 5% CO_2._

**siRNA library screening.** We developed an RNA interference library comprising 22 genes that encode common epigenetic modifiers, including writers and erasers. Each gene in the library was targeted by three distinct siRNA sequences. The siRNAs targeting these genes were combined and transfected into three different HCC cell lines, namely Huh7, HepG2, and PLC/PRF/5. After a 24-hour incubation, the cells were seeded in 96-well plates at a density of 4000 cells per well. Once adhered, the cells were exposed to oxaliplatin for 48 hours. Relative viability were subsequently determined by assessing cell viability using a CCK-8 assay.

**Xenograft models.** NOD/SCID mice (6-week-old, 18-22 g, male) were purchased from Vital River Laboratory Animal Technology Co., Ltd. (Beijing, China) and housed in specific pathogen-free (SPF) conditions. The mice were randomly assigned to experimental groups according to the study design without blinding. Each group involves six animals. Huh7 cells stably transfected with shMETTL3 lentivirus or its negative control were prepared by digestion and subsequent washing. Subsequently, 5 x 10^6^ cells were injected subcutaneously into the axilla of the mice to establish subcutaneous tumors. Tumor growth was monitored every 5 days by measuring the length (L) and width (W) using calipers. The tumor volume (V) was calculated using the formula V = (L x W^2^)/2. Approximately four weeks after tumor inoculation, the mice were euthanized, and the tumors were excised, weighed, photographed, and fixed for further analysis.

The C57BL/6J mice (8-week-old, 18-22 g, male) were obtained from Vital River Laboratory Animal Technology Co., Ltd and housed under SPF conditions. For the orthotopic transplantation model, 24 animals were randomly allocated to groups, with each group consisting of six animals without blinding. A mixture of 5 x 10^5^ Hepa 1-6-luc cells (mouse hepatoma cell with luciferase stable expression) and Matrigel (Corning， USA) was injected into the left lobe of the mice’s livers via laparotomy. 24 days after surgery, the mice were photographed using the *in vivo* imaging system IVIS Lumina spectrum (Perkin Elmer, USA) with D-luciferin (Gold Biotechnology, USA) as the substrate. Subsequently, the mice were euthanized, and their livers were removed, imaged and fixed for further analysis.

To investigate the potential clinical benefits of targeting METTL3, we utilized shMETTL3 or the specific inhibitor STM2457 in combination with OXA in both subcutaneous and orthotopic models. In the subcutaneous model, ten days after cell implantation, OXA solution (diluted in 5% glucose injection) or the vehicle was administrated via intraperitoneal injection (i.p.) to the mice carrying Huh7-shNC or Huh7-shMETTL3 cells without blinding. The dosage of OXA was 5 mg/kg, and it was given every 3 days for a period of 3 weeks. In the orthotopic model, the mice carrying Hepa 1-6 cells were randomly divided into four groups. Starting from the fifth day after surgery, OXA solution was administered to the mice as described earlier, with or without STM2457 administration. STM2457 was dissolved in DMSO and further diluted with corn oil. A dosage of 30 mg/kg STM2457 or the vehicle was delivered via i.p. injection every other day for 2 weeks. All animal experiments were conducted in accordance with the specific animal treatment guidelines and were approved by the animal management committee of Sun Yat-sen University Cancer Center.

**HCC organoids construction.** The fresh HCC tissue was minced into small pieces, rinsed and dissociated with tumor dissociation kit (BioGenous, China) at 37 ℃ for 60-120 min. Digestion was stopped by adding 2% BSA-PBS solution and the cells were filtered through a 70 μm Nylon cell strainer, followed by centrifuging (5 min at 300 g). Cells were resuspended in the Basement Membrane Matrix for Organoid Culture (MedChemExpress, USA) according to the volume of pellets, and seeded into 48-well plates. The plate was warmed in the incubator for 10 min to allow the Matrigel to solidify. Then Hepatocellular Carcinoma Organoid Culture Medium (BioGenous, China) was added to the wells. After three successful passages, the organoids were either preserved or used for further assays.

**Drug sensitivity test of organoids.** The organoids were harvested from Matrigel and dissociated using Organoid Dissociation Solution (BioGenous, China). Following cell counting, the organoids were resuspended in Matrigel and seeded at approximately 2000 cells per well in 96-well Flat Clear Bottom White Microplates (Beyotime, China). After several days of culture, STM2457 and oxaliplatin were added to the cells either individually or in combination at various concentrations for a duration of five days. On day 5, cell viability was assessed using the Organoid ATP Viability Assay Kit (AbsinBioscience, China). Additionally, bright field images of the cells were captured on day 0 and day 5. To assess the effect of drug combination treatments, combination index (CI) values were calculated based on a 3 x 2 dose-response matrix and analyzed with CalcuSyn software. CI values less than 1 were considered indicative of synergistic interactions.

**3D Immuno-Imaging of organoids.** The organoid staining was performed following established protocols with slight modifications. In brief, organoids were harvested from Matrigel and fixed with 4% paraformaldehyde (PFA) at 4°C for 1 hour. After centrifugation, the pellet was resuspended in 0.2% Triton-X for 1 hour to permeabilize the cells. For blocking, the organoids were incubated in a 5% BSA solution for 1 hour. Primary antibodies (METTL3, 1:200, Proteintech; HepPar-1, 1:200, ZHONGSHAN GOLDEN BRIDGE BIOTECHNOLOGY; Arg1, 1:200, Proteintech) were then added, and the organoids were incubated at 4°C overnight. Following three washes, the organoids were incubated with fluorescently labeled secondary antibodies for 2 hours at room temperature. After additional washes, the pellets were resuspended in antifade mounting medium containing DAPI (Beyotime, China). A proper area was marked in the center of a slide using a PAP pen, and adhesive tape was placed on both sides of the marked area. The organoid suspension was placed within this area and covered with a coverslip. The prepared slides were then observed and imaged using confocal microscopy (Carl Zeiss AG, German) with the Z-stack mode.

**Immunoblotting.** The cells were washed twice with PBS and then lysed by RIPA buffer (Invitrogen, USA) supplemented with protease inhibitor (Roche, USA). The lysate was centrifuged at the highest speed at 4℃, and the supernatant was collected for quantification by bicinchoninic acid (BCA) Kit (Invitrogen, USA). After normalization and preparation, the protein extracts were separated by 8-12% SDS-PAGE and transferred onto polyvinylidene fluoride (PVDF) membranes (Roche, USA). The membranes were then incubated overnight at 4℃ with specific high-affinity primary antibodies, including anti-METTL3 antibody (1:1000, Proteintech Group, USA), anti-TRIM21 antibody (1:1000, Proteintech Group, USA), anti-γH2AX antibody (1:1000, CST, USA), and anti-G6PD antibody (1:2000, Abcam, USA). Subsequently, the membranes were incubated with peroxidase (HRP)-conjugated secondary antibodies at room temperature for 1 h, followed by chemiluminescence detection. Additional information about primary antibodies can be found in Supplementary Table 1.

**Immunohistochemistry (IHC).** IHC was performed following previously described methods[1]. Tissue microarrays containing samples from 212 HCC patients were utilized to assess the expression levels of METTL3 and G6PD. In the case of mice, the tissues were promptly excised and fixed with 10% formaldehyde/PBS for subsequent histological analysis. In brief, tissue sections were blocked and then incubated overnight at 4°C with primary antibodies. Then the sections were incubated with the anti-rabbit biotinylated secondary antibody and peroxidase-conjugated avidin-biotin complex (Dako, Mississauga, Canada). Visualization of staining was achieved using 3,3′-diaminobenzidine (Dako). The staining intensity was scored as follows: 0 for negative; 1 for weak; 2 for moderate; and 3 for strong. The frequency of positive cells was graded as follows: 0 for less than 5%, 1 for 5% to 25%, 2 for 26% to 50%, 3 for 51% to 75%, and 4 for greater than 75%. The final score for a specific protein was calculated as the product of the intensity and frequency scores.

**Edu assay.** The Edu proliferation assay was conducted following the instructions provided by the manufacturer (RIBOBIO, C10310). Briefly, the cells were seeded in 12-cell plates and allowed to attach for 24 h before being exposed to Edu solution for 2 h. Subsequently, the cells were washed, fixed with 4% paraformaldehyde, and permeabilized with Triton X-100. After that, the cells were incubated with 1 x Apollo solution for 30 min, followed by a thorough washing step. Finally, the cells were stained with Hoechst33342 and visualized using a fluorescence microscope, and images were captured.

**NADPH quantification.** The quantification of NADPH was performed using NADP+/NADPH Quantification kit (Biovision, USA) according to the manufacturer’s instructions. Briefly, NADP+ was converted into NADPH, and only NADPH (not NAD+ or NADH) could reduce WST-8 to form formazan. The formation of formazan was quantified by colorimetry at 450 nm.

**ROS detection.** Intracellular ROS levels were assessed following the method described previously [2]. Briefly, cells were incubated with DCFH-DA (Beyotime, China) for 30 min. After incubation, cells were washed, digested, and harvested. The levels of intracellular ROS were measured using flow cytometry (Beckman, USA) with the channel FL1.

**G6PD activity detection.** G6PD enzyme activity was determined with the kit following the manufactures’ instructions (S0189, Beyotime, Shanghai, China). Cells were harvested and lysed with extraction buffer. After centrifugation, the supernatant was used for the detection. Standards or samples were added to the detection mixture, which included reaction buffer and chromogenic solution, in 96-well plate. The plate was incubated at 37℃ for 10 min, and the absorbance of the samples was measured at 450 nm using a Microplate Reader. A standard curve was constructed using a series of diluted standards, and the G6PD enzyme activity of the sample was calculated based on the curve.

**Cell proliferation and colony formation assay.** For proliferation assay, cells were seeded into 96-well plates with a density of 3000 cells per well. Cell viability was measured at different time points using the cell counting kit-8 (CCK8) reagent (MedChem Express, USA) according to the manufacturer’s instructions.

For colony formation assay, the cells were seeded into 6-well plates with a density of 500-1000 cells per well and cultured in complete medium for approximately two weeks. Once visible colonies had formed, the cells were washed with PBS and fixed with methanol for 15 minutes. Subsequently, they were stained with 0.1% crystal violet for 30 minutes. The plates were then washed, dried, and imaged for further analysis.

**Cell cycle.** Cell cycle analysis was performed as follows: 1 × 10^6^ cells were collected, washed and fixed with 75% cold ethanol for 24 h at 4 °C. After washed with PBS twice, the cells were stained with propidium iodide and RNase A (Keygen, China) at room temperature for 30 min. The samples were assessed using an ACEA NovoCyte flow cytometer (Agilent, USA), and the distribution of cell cycle was analyzed using ACEA NovoExpress software.

**Protein stability.** After treated with 50 μg/mL cycloheximide (MedChem Express, USA), the cells were collected at different time points and subjected to western blot assay. The WB images were analyzed using ImageJ software, and the results were summarized and presented in a linear graph.

**Untargeted metabolomics profiling.** Untargeted metabolomics profiling was conducted on the XploreMET platform (Metabo-Profile, Shanghai, China) following previously published methods with minor modifications [3]. In brief, frozen samples were mixed with pre-chilled zirconium oxide beads (25 mg) and internal standard (10 μL) before 50 μL of 50% pre-chilled methanol was added for automated homogenization with the BB24 (Next Advance, Inc., USA). The resulting supernatant was collected through high-speed centrifugation (14,000 × g, 4 °C, 20 min) and combined with pre-chilled methanol/chloroform (v/v = 3/1, 175 μL). This mixture underwent additional high-speed centrifugation (14,000 × g, 4°C, 20 min) to collect the supernatant. The resulting 200 μL supernatant was transferred to an autosampler vial, while the residual supernatant was pooled to generate quality control samples. The samples were dried using the FreeZone freeze dryer (Labconco, Kansas City, MO) and then derivatized with 50 μL of methoxyamine (20 mg/mL in pyridine) at 30°C for 2 hours. Subsequently, 50 μL of MSTFA (1% TMCS) containing FAMEs as retention indices were added at 37.5°C for 1 hour using the sample preparation head. The derivatized specimens were then injected via the sample injection head. A Pegasus HT GC-TOF/MS system (Leco Corp, USA) equipped with an Agilent 7890B gas chromatograph was used for GC-TOFMS analysis. The resulting raw data were processed using iMAP 1.0 and transformed into comparable data sets to facilitate further statistical analysis. Statistical significance was determined using the Student t-test, with a calculated fold change of 1.5 or a p-value of 0.05 chosen as the threshold for significance.

**Clinical specimens.** HCC tissues and adjacent liver tissues, identified by two independent pathologists, were collected from patients who provided informed consent in Sun Yat-sen University Cancer Center between 2016 to 2020. The usage of these specimens was approved by the ethics committee of Sun Yat-sen University Cancer Center. The specimens obtained from patients were used in the analysis of the relationship between METTL3 expression and OXA efficacy.

**Plasmids and transfection.** METTL3 knockdown lentivirus plasmids (shMETTL3-1, shMETTL3-2) and their corresponding control were purchased from GeneCopoeia Co., Ltd. (Guangzhou, China). In addition, a METTL3 overexpression lentivirus plasmid was constructed by inserting the CDS sequence into the pLVX vector, and the accuracy of the inserted sequence was confirmed by Sanger sequencing. The virus particles were produced, collected, and filtered using 293FT cells (Invitrogen, USA) and 3^rd^ generation lentivirus packaging plasmids (Addgene, USA). For each 6-cm dish of cells at 60% confluence, 1 mL virus was added along with 8 μg/mL polybrene (Sigma). After selection with puromycin, the knockdown efficiency was assessed by western blot analysis.

**RNA extraction and quantitative real-time PCR.** Total RNA was lysed by Trizol reagent (Invitrogen, USA) according to the manufacturer’s protocol. Subsequently, cDNA was synthesized using PrimeScript RT reagent Kit (Takara, Japan). For qRT-PCR, the iTaq Universal SYBR Green supermix (Bio-Rad, USA), 96-well white PCR plate and the LightCycler 480 system (Roche, USA) were employed. All primers used in the assay were synthesized by Ruibio Biotech company (Beijing, China). Some primers were derived from PrimerBank (https://pga.mgh.harvard.edu/primerbank/).

**RNA Immunoprecipitation.** RIP assay was conducted using EZ-Magna RIP Kit (Millipore, USA) according to the manufacturer’s protocol. Briefly, a total of 2 x 10^7^ cells were lysed, and the lysates were immunoprecipitated with either rabbit IgG or specific primary antibodies overnight at 4℃. Following washing steps, the beads-protein-RNA complex was subjected to protease K digestion at 55℃ for 30min, followed by precipitating with sodium acetate (pH 5.2) and absolute alcohol. After purification, the protein-bound RNA was reverse transcribed and then analyzed by RT-PCR.

**Methylated RNA Immunoprecipitation-PCR (MeRIP-PCR).** The assay was performed with some modifications as previously described [4]. In brief, RNA was extracted using Trizol reagent. mRNA purification was performed using the Hydrophilic Streptavidin Magnetic Beads (S1421S, NEB, USA) and biotin-(dT)18 (S1325S, NEB). The purified mRNA was subsequently fragmented using metal ions (E6150S, NEB) at 94℃ for 3 min and further purified using a column-based method (Qiagen, USA). The resulting fragments were incubated with magnetic Dynabead-bound anti-m^6^A antibody (202003, Synaptic Systems, Germany) at 4℃ for 2 h. The beads complex was then digested with protease K at 55℃ for 30 min and purified with the RNeasy MinElute Cleanup Kit (Qiagen). The immunoprecipitated RNA was reverse-transcribed (Takara, Japan). The level of methylation at different regions of TRIM21 mRNA was subsequently detected using eight pairs of primers that covered the full length of TRIM21.

**RNA stability assays:** Cells were treated with actinomycin D at a final concentration of 5 μg/ml. Total RNA was extracted at 0, 2, 4, 6 and 8 hours after adding actinomycin D. The mRNA stability was determined by RT-qPCR and the data of TRIM21 were normalized to ActD-resistant U6 RNA as a stable internal control.

**Cytotoxicity assays.** Cell counting Kit-8 (CCK-8) was used to quantify the cytotoxicity induced by the drug. Cells, both with or without METTL3 silencing, were seeded in 96-well plates and incubated for 24 hours. Subsequently, the cells were then exposed to the graded concentrations of OXA for 48 hours, followed by treatment with CCK-8 for the assessment of OXA sensitivity. To evaluate the in vitro synergy of combinations of OXA with DHEA or STM2457, both agents were administered simultaneously for 48 hours at varying concentrations, followed by evaluating via CCK8 assay. Or the treatments were applied on colonies for at least ten days, followed by Crystal violet staining and photography. The data for each treatment condition (single drugs and combinations) were put into the CalcuSyn software. The combination index (CI) was calculated based on the inhibition rates across different dose groups. The software uses the median-effect principle of the Chou-Talalay method to calculate the CI values. Additionally, the fraction affected (Fa)/CI plot was used to determine synergy or antagonism. A CI value below 1 indicates synergism between the two agents, while a value above 1 suggests antagonism; a CI of 1 signifies an additive effect.

**Dot blot.** As described previously[5], mRNA was denatured at 95℃ for 3 min and then diluted in a serial manner. Subsequently, the diluted samples were spotted onto Hybond N+ membranes (GE Healthcare) and dried before being crosslinked using crosslinker (Spectroline, USA). Following crosslinking, the membranes were washed, blocked, and incubated with m^6^A antibody overnight at 4℃. After incubation with a secondary antibody, the signal was detected using ECL with ChemiDoc imaging system (Bio-Rad, USA). Additionally, the membranes were stained with 0.01% methylene blue and scanned to quantify the amount of RNA for further analysis.

**DNA-platination detection with ICP-MS.** Cells were exposed to 20 μM oxaliplatin for 3 h at 37 ℃. Genomic DNA was then extracted and purified using a silica-based column DNA purification kit (TIANGEN, China) according to the manufacturer’s instructions. DNA concentration was measured at 260 nm using a Nanodrop. The DNA was subsequently treated with an equal volume of reverse aqua regia (HNO₃: HCl = 3:1) and heated for 1 hour. Samples were analyzed at the Instrumental Analysis & Research Center of Sun Yat-sen University using an iCAP Q (Thermo Fisher Scientific, USA). The platinum concentration was normalized to the DNA amount.

**Statistical analysis.** The data were analyzed using GraphPad Prism software (V8) and presented as the mean ± standard error of the mean (SEM) with at least three independent experiments. P values (*P < 0.05 and **P < 0.005) were calculated by paired and two‐tailed t‐test. Pairwise differences were assessed using a two-tailed Student's t-test. Survival analysis was performed using Log-rank Mantel-Cox testing. For other data, one-way ANOVA with Tukey’s multiple comparison test was utilized. Statistical significance was considered at a *P*-value of less than 0.05 in all cases.

**REFERENCES**

1. Cai M, Hou J, Rao H, Luo R, Li M, Pei X*, et al.* High expression of H3K27me3 in human hepatocellular carcinomas correlates closely with vascular invasion and predicts worse prognosis in patients. *Molecular medicine (Cambridge, Mass).* 2011; 17, 12-20.

2. Ju HQ, Lu YX, Wu QN, Liu J, Zeng ZL, Mo HY*, et al.* Disrupting G6PD-mediated Redox homeostasis enhances chemosensitivity in colorectal cancer. *Oncogene.* 2017; 36, 6282-6292.

3. Wang X, Zhang X, Cao K, Zeng M, Fu X, Zheng A*, et al.* Cardiac disruption of SDHAF4-mediated mitochondrial complex II assembly promotes dilated cardiomyopathy. *Nat Commun.* 2022; 13, 3947.

4. Meyer K, Saletore Y, Zumbo P, Elemento O, Mason C, Jaffrey S. Comprehensive analysis of mRNA methylation reveals enrichment in 3' UTRs and near stop codons. *Cell.* 2012; 149, 1635-1646.

5. Li E, Xia M, Du Y, Long K, Ji F, Pan F*, et al.* METTL3 promotes homologous recombination repair and modulates chemotherapeutic response in breast cancer by regulating the EGF/RAD51 axis. *eLife.* 2022; 11, e75231.

**Supplementary figures and legends**


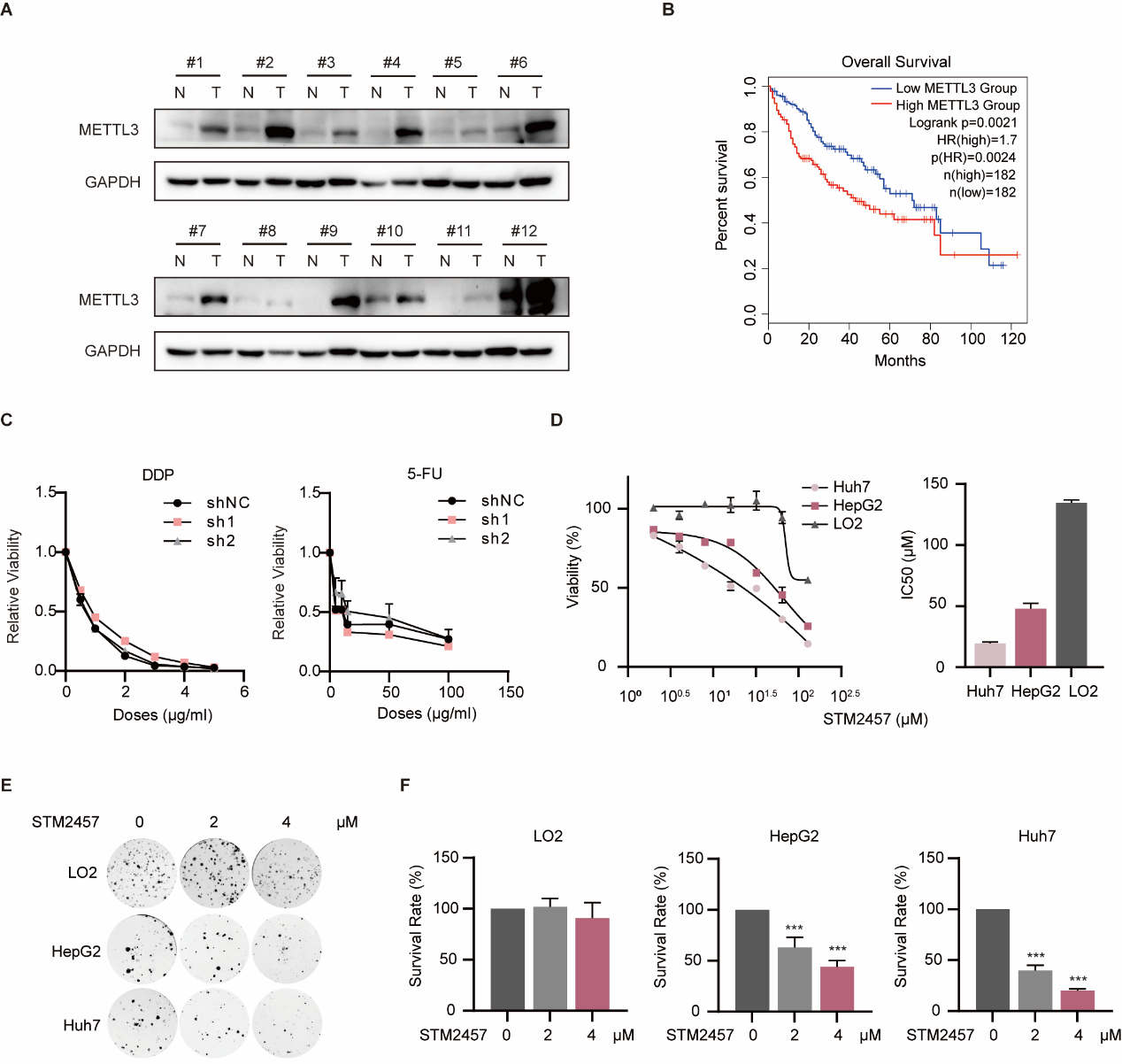


**Figure S1. Expression and impact of METTL3 in HCC.** (A) Western blot analysis revealing the expression levels of METTL3 in 12 pairs of frozen HCC tissues. (B) Kaplan-Meier survival analysis illustrating the overall survival (OS) trends based on METTL3 expression in HCC patients, using data from TCGA database. (C) Assessment of relative cell viability upon exposure to varying concentrations of DDP or 5-FU. (D) Assessment of cell viability in Huh7, HepG2, and LO2 cell lines upon exposure to varying concentrations of STM2457, with determination of IC50 values (right panel). (E) Colony formation assays depicting the cellular survival following exposure to diverse concentrations of STM2457, accompanied by calculation of relative survival rates (F).


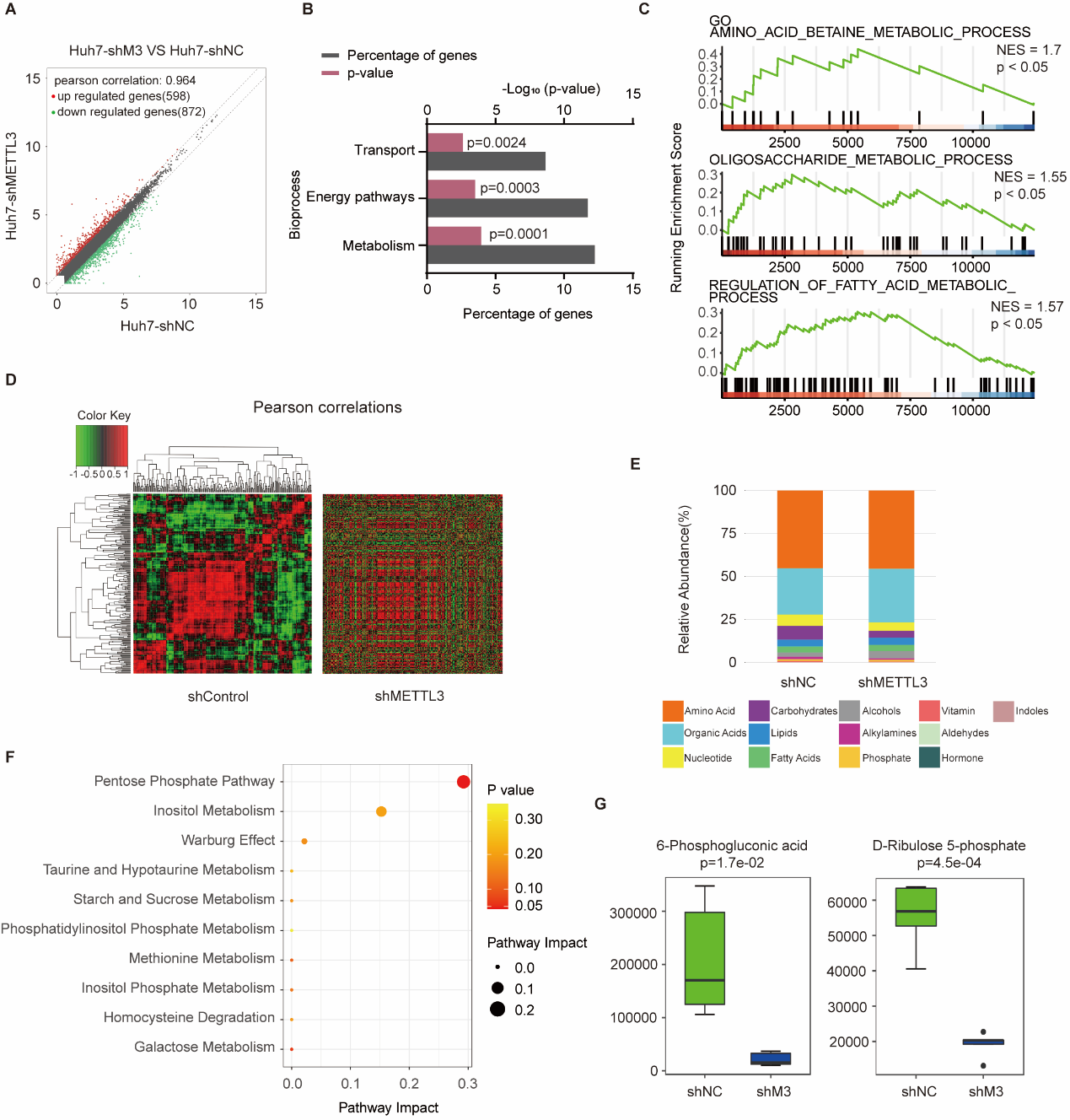


**Figure S2. Influence of METTL3 silencing on HCC cell metabolism.** (A) Scatter plot illustrating the differential gene expression profile identified through RNA-seq analysis following METTL3 silencing in Huh7 cells. (B) Enrichment analysis highlighting differential biological processes identified through RNA-seq data using the Funrich tool. (C) Gene Set Enrichment Analysis (GSEA) visualization depicting the alteration of metabolism-related genes in METTL3-knockdown cells. (D) Pearson correlation heatmaps demonstrating the correlation patterns of metabolites between the shMETTL3 and control groups. (E) Comparison of the relative abundance of distinct metabolite groups between experimental groups. (F) Bubble map displaying the enriched metabolic pathways identified through analysis of differential metabolites. (G) Comparison of the average levels of 6-phosphogluconic acid and D-ribulose 5-phosphate between the control and shMETTL3 groups.


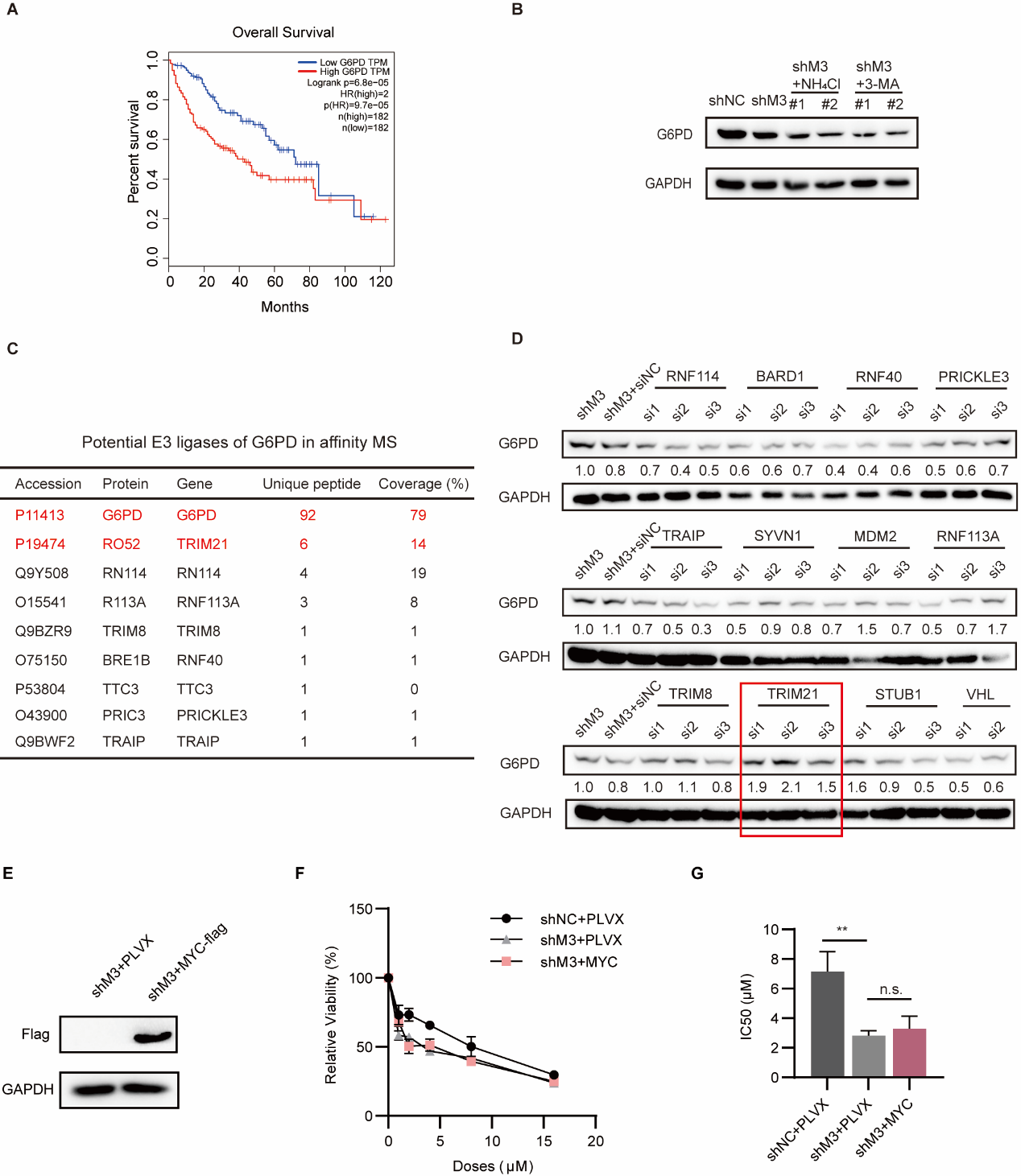


**Figure S3. METTL3-mediated regulation of G6PD via the ubiquitin-proteasome pathway.** (A) Kaplan-Meier analysis illustrating the overall survival outcomes for 364 HCC patients categorized based on G6PD expression levels using data from TCGA database. (B) Expression levels of G6PD and GAPDH analyzed in shNC and shMETTL3 cells, with or without treatment of lysosome inhibitors. (C) Identification of potential E3 ligases associated with G6PD through anti-FLAG co-immunoprecipitation (co-IP) followed by mass spectrometry (MS). (D) Construction of a siRNA library targeting potential G6PD-associated E3 ligases sourced from affinity MS, UbiBrowser database, and relevant literatures. Analysis of G6PD and GAPDH expression in shMETTL3 cells transfected with specific siRNAs, with relative gray values of G6PD/GAPDH normalized to shMETTL3 cells. (E) The efficiency of MYC-flag overexpression verified at protein levels via WB. (F) The relative cell viability with different doses of OXA treatment for 48 h detected via CCK-8 assays with the IC50 values calculated (G).


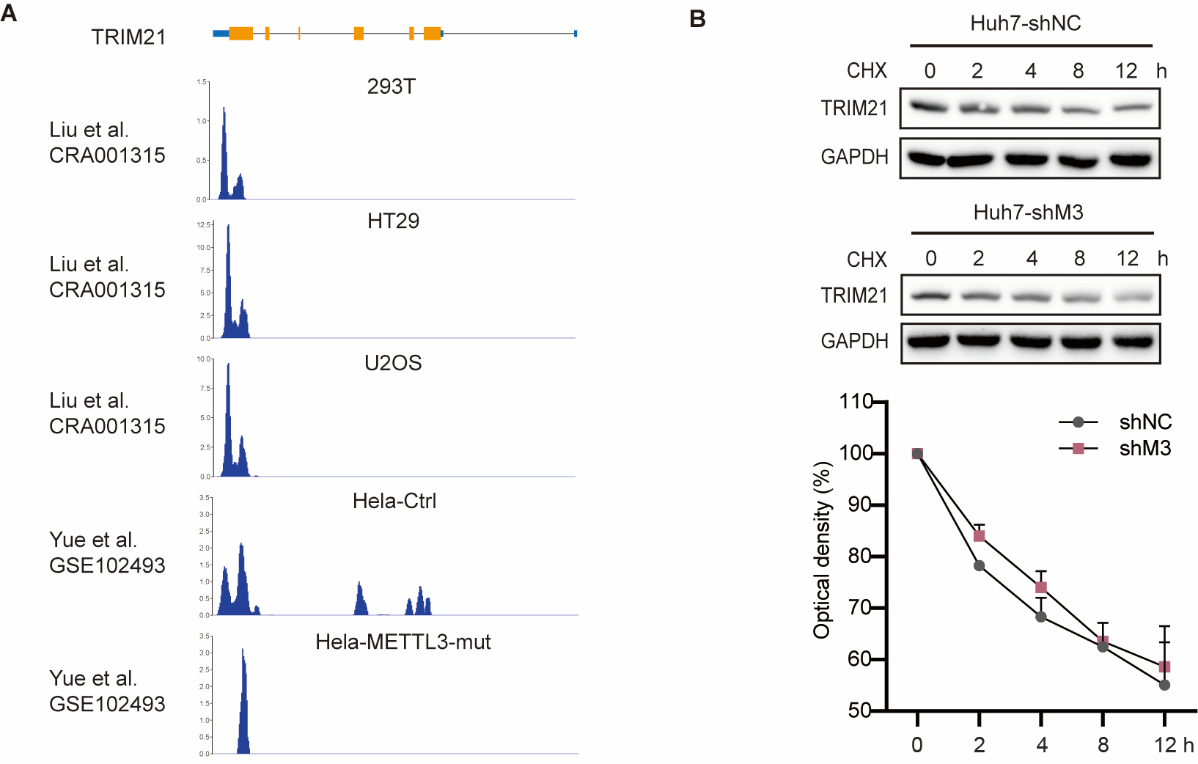


**Figure S4. METTL3-mediated regulation of TRIM21 mRNA stability via an m^6^A-dependent mechanism.** (A) Assessment of m^6^A modifications on TRIM21 mRNA in diverse model cells sourced from the RMVar database. The impact of METTL3 mutation in Hela cells on m^6^A modifications of TRIM21 is demonstrated. (B) Western blot analysis depicting the protein levels of TRIM21 and GAPDH in Huh7-shNC and shMETTL3 cells treated with cycloheximide (CHX) at various time intervals (upper panel). Quantitative analysis of the results is presented in the lower panel.


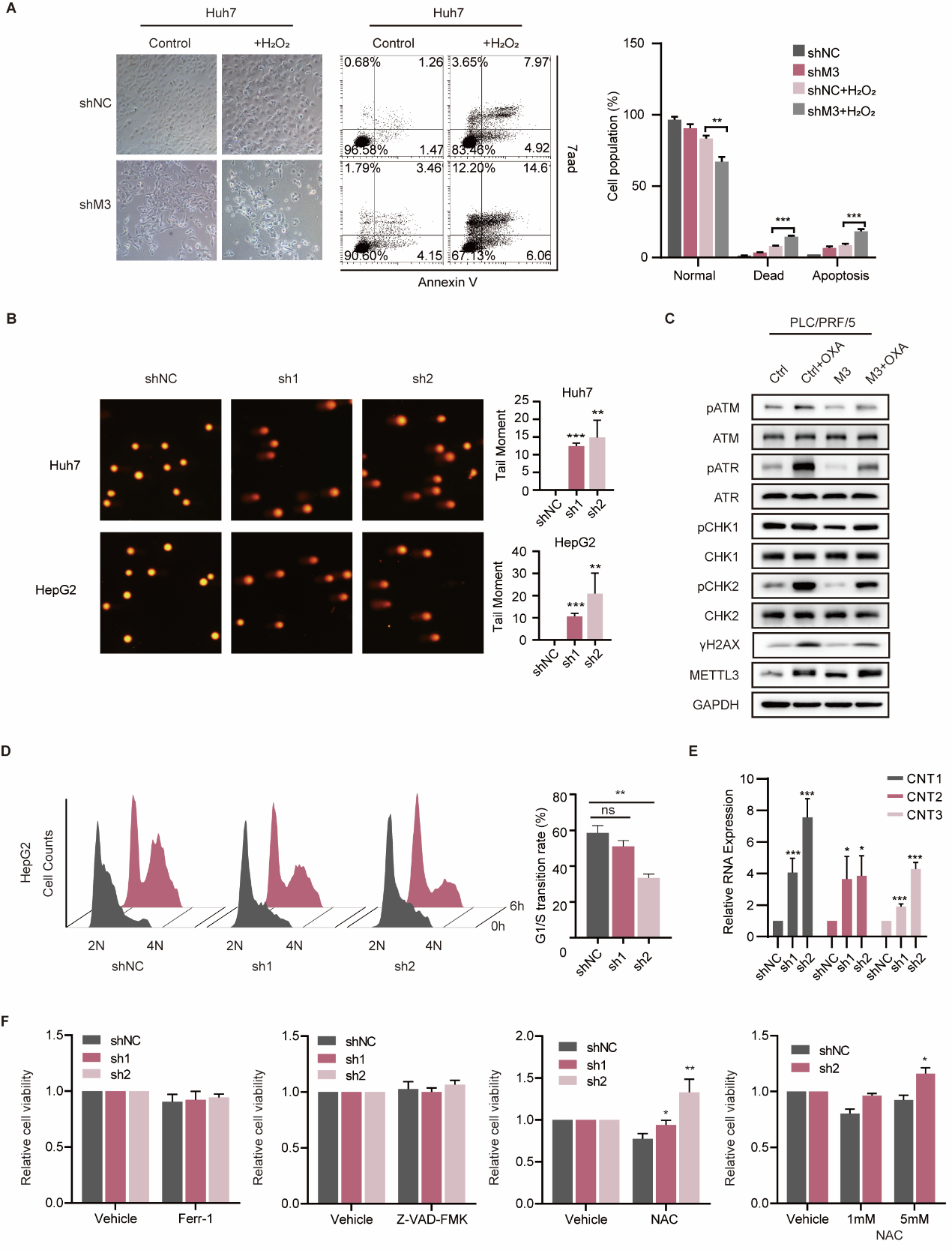


**Figure S5. METTL3-mediated regulation of HCC cell cycle and redox state.** (A) Evaluation of the sensitivity of Huh7-shNC and Huh7-shMETTL3 cells to oxidative stress. Left panel showcases cell morphology under H_2_O_2_ treatment. Cell apoptosis assessed by FACS using Annexin V/7aad staining is depicted in the middle panel. Quantitative representation of distinct cell populations is presented in the right panel. (B) Detection of DNA damage subsequent to METTL3 silencing in Huh7 and HepG2 cells through comet assays (left panel). Quantification of tail moment values is provided in the right panel. (C) Detection of proteins in ATM-CHK2/ATR-CHK1 DNA damage response pathways in METTL3 overexpression and control cells with or without oxaliplatin treatment. (D) Cell cycle progression analyses displaying G1/S transition facilitated by thymidine-induced synchronization (left panel). Statistical summary of G1/S transition rates is shown in the right panel. (E) Relative RNA expression of CNTs following METTL3 silencing detected via qPCR. (F) Assessment of cell viability using CCK-8 assays, in conjunction with treatment involving Ferr-1 (ferroptosis inhibitor), Z-VAD-FMK (apoptosis inhibitor), and NAC (antioxidant).


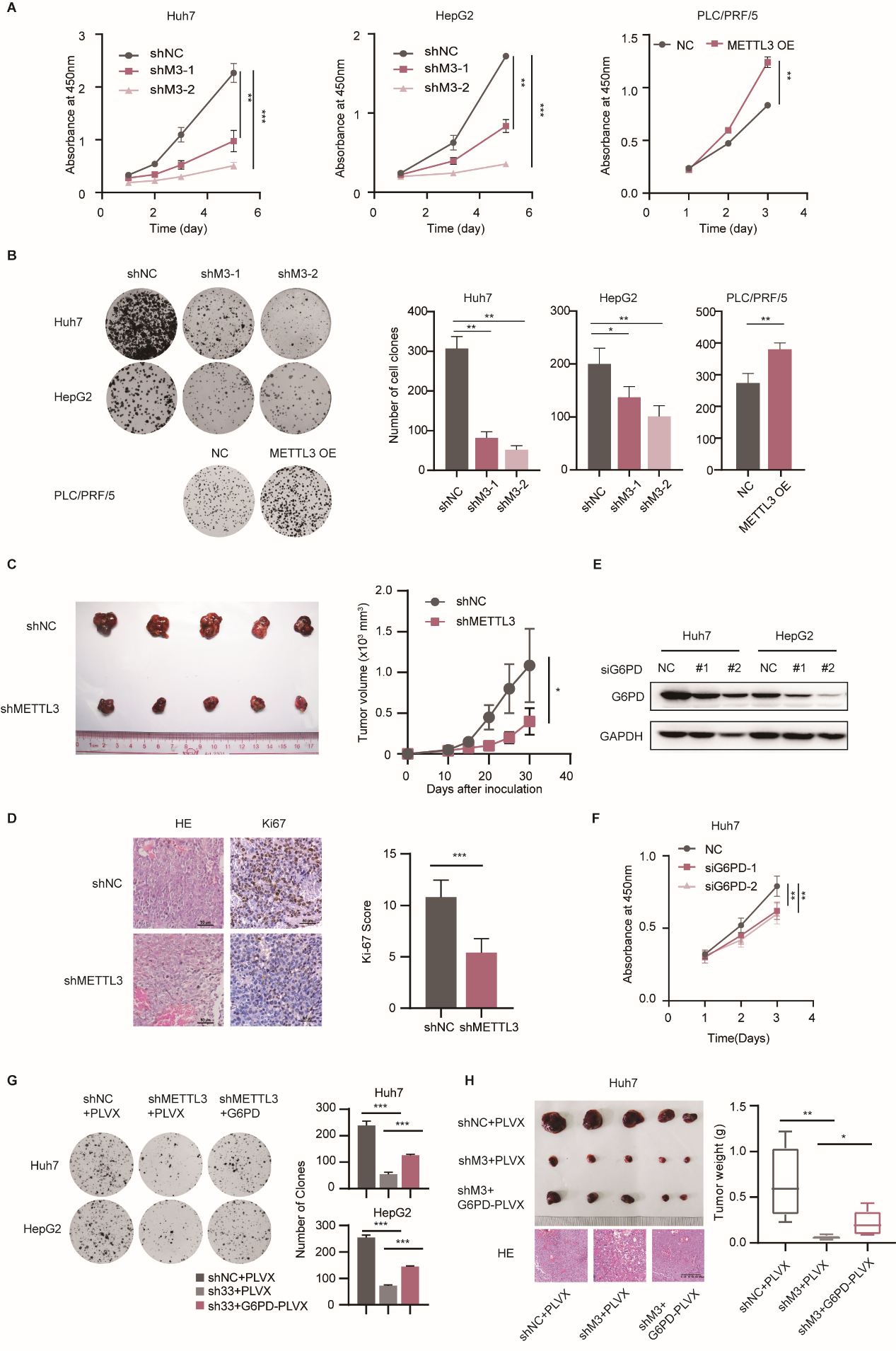


**Figure S6.** **METTL3 regulated HCC proliferation *in vitro and in vivo*.** (A) Proliferation of Huh7, HepG2, and PLC/PRF/5 cells under METTL3 disturbance determined through CCK8 proliferation assays. (B) Colony formation assays revealing the impact of METTL3 disturbance on proliferation of Huh7, HepG2, and PLC/PRF/5 cells (left panel). Quantitative representation of results is shown in the right panel. (C) HCC subcutaneous tumor growth in nude mice achieved by silencing METTL3 (*n*=5, left panel). Growth curve depicting tumor volume monitored over 5-day intervals is presented in the right panel. (D) Representative images depicting HE and Ki-67 immunohistochemistry in subcutaneous tissue sections (left panel) with Ki-67 score statistics provided in the right panel. (E) Validation of G6PD knockdown efficiency at the protein level via Western blot. (F) Assessment of HCC cell proliferation upon G6PD silencing using CCK8 assays. (G) Colony formation assays showcasing cell proliferation of control and METTL3-silenced cells with or without G6PD overexpression. The statistics was shown in the right panel. (H) Subcutaneous mouse tumor models comparing tumors from control and shMETTL3 Huh7 cells, with or without G6PD overexpression. The statistics of tumor weights was shown in the right panel. Data are presented as mean ± SD (**p* < 0.05, ***p* < 0.01, and ****p* < 0.001).


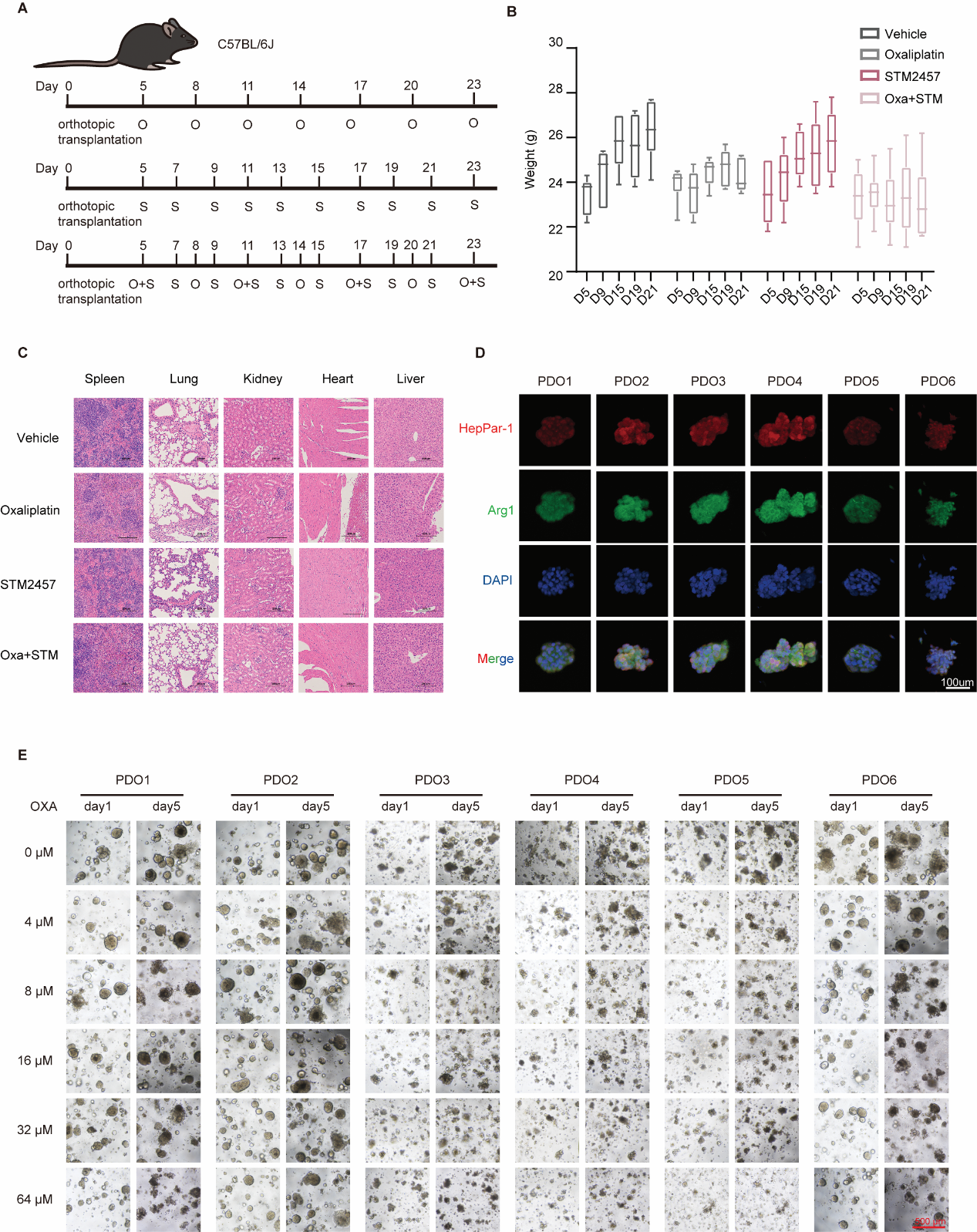


**Figure S7. Effect of STM2457 on mice and organoids.** (A) Schematic representation of treatment regimens for distinct groups of orthotopic C57BL/6J mice. (B) Monitoring of body weights across varied groups of orthotopic C57BL/6J mice. (C) HE staining of specified organs extracted from orthotopic C57BL/6J mice. (D) Identification of HCC organoids via HepPar-1 and Arg1 double immunofluorescence staining. (E) Bright field of HCC organoids treated with different doses of OXA.
