# Supplementary material for: METTL3 confers oxaliplatin resistance through the activation of G6PD-enhanced pentose phosphate pathway in hepatocellular carcinoma
Source: Cell Death Differ. 2024 Oct 29;32(3):466–79. doi: 10.1038/s41418-024-01406-2 (PMC11894169; doi:10.1038/s41418-024-01406-2)
Supplement: Supplementary file 2 — Supplementary tables [file 41418_2024_1406_MOESM2_ESM.docx]

**Supplementary Tables**

Supplementary Table 1. Primary antibodies used in this study

| Antibody name | Corporation | Catalog | Application |
| --- | --- | --- | --- |
| METTL3 | Proteintech | 15073-1-AP | WB, IP |
| G6PD | Abcam | Ab210702 | WB |
| TRIM21 | Proteintech | 12108-1-AP | WB |
| Anti-m^6^A | sysy | 202003 | IP, dotblot |
| H6PD | Abcam | Ab170895 | WB |
| PGLS | Abclonal | A8366 | WB |
| ATM | CST | 2873 | WB |
| ATR | CST | 2790 | WB |
| p-ATM | CST | 13050 | WB |
| p-ATR | CST | 30632 | WB |
| γH2AX | CST | 9718 | WB, IF |
| p-CHK2 | CST | 2197 | WB |
| CHK2 | CST | 6334 | WB |
| Ki-67 | CST | 34330 | IHC |
| Cleaved-caspase 3 | CST | 9664 | IHC |
| GAPDH | Proteintech | 60004-1-Ig | WB |
| ubiquitin | CST | 20326 | WB |
| YTHDF2 | Proteintech | 24744-1-A | WB, RIP |
| FLAG | CST | 14793 | WB |
| Myc | CST | 2272 | WB |
| HA | Abmart | M20003 | WB |
| CDC25A | Proteintech | 55031-1-AP | WB |
| CDK2 | Proteintech | 10122-1-AP | WB |
| CDK2 T14 | Abcam | ab68265 | WB |
| Arg1 | Proteintech | 16001-1-AP | IF |
| HepPar-1 | ZHONGSHAN GOLDEN BRIDGE BIOTECHNOLOGY | ZM-0131 | IF |

Supplementary Table 2. Inhibitors or reagents used in the study

| Antibody name | Corporation | Catalog | Application |
| --- | --- | --- | --- |
| MG132 | MCE | HY-13259 | Proteasome inhibitor |
| 3-Methyladenine | MCE | HY-19312 | Autophagy inhibitor |
| Cycloheximide | MCE | HY-12320 | Protein synthesis inhibitor |
| STM2457 | TOPSCIENCE | T9060 | METTL3 inhibitor |
| oxaliplatin | MCE | HY-17371 | Chemotherapeutic agent |
| DHEA | MCE | HY-14650 | G6PD inhibitor |
| D-Luciferin Potassium Salt | bioWORLD | 115144-35-9 | *In vivo* imaging |
| Thymidine | MCE | HY-N1150 | Cell cycle inhibitor |
| CCK-8 | MCE | HY-K0301 | Detecting cell viability |

Supplementary Table 3. The target sequences of shRNA and siRNA.

| **Name** | **Sequence** |
| --- | --- |
| METTL3 | sh#1 5’- GCACTTGGATCTACGGAATCC -3’ |
|  | sh#2 5’- GGAGATCCTAGAGCTATTAAA -3’ |
|  | si#1 5’- CAAGTATGTTCACTATGAA -3’ |
|  | si#2 5’- GACTGCTCTTTCCTTAATA -3’ |
|  | si#3 5’- GGACTCGACTACAGTAGCT -3’ |
| TRIM21 | si#1 5’- GCAGCACGCTTGACAATGA -3’ |
|  | si#2 5’- GGACAATTTGGTTGTGGAA -3’ |
| NSUN3 | si#1 5’- GGCAGACGTTGGAATCTTT -3’ |
|  | si#2 5’- GATCGAAGCTGGTTGTTTT -3’ |
|  | si#3 5’- GGCCGAATCCCTTCAGAAA -3’ |
| HDAC1 | si#1 5’- GCGACTGTTTGAGAACCTT -3’ |
|  | si#2 5’- GGGATCGGTTAGGTTGCTT -3’ |
|  | si#3 5’- GGCCGAATCCCTTCAGAAA -3’ |
| HDAC2 | si#1 5’- CCGTAATGTTGCTCGATGT -3’ |
|  | si#2 5’- GACCCATAACTTGCTGTTA -3’ |
|  | si#3 5’- AACCGACAACAGACTGATA -3’ |
| DNMT1 | si#1 5’- GAAGAGACGTAGAGTTACA -3’ |
|  | si#2 5’- GGAACTTTGTCTCCTTCAA -3’ |
|  | si#3 5’- CAATGAGACTGACATCAAA -3’ |
| PRMT1 | si#1 5’- GCAACTCCATGTTTCATAA -3’ |
|  | si#2 5’- AGACGGTGTTCTACATGGA -3’ |
|  | si#3 5’- CCATCGACCTGGACTTCAA -3’ |
| PRMT5 | si#1 5’- GCACCAGTCTGTTCTGCTA -3’ |
|  | si#2 5’- GAGGTGCAGTTCATCATCA -3’ |
|  | si#3 5’- TGGACAATCTGGAATCTCA -3’ |
| KDM2A | si#1 5’- GCAAGCAGATCACTCGAAA -3’ |
|  | si#2 5’- GCACCATGGTACGGGAAAA -3’ |
|  | si#3 5’- GGTGGGCAGTAGGAATCAA -3’ |
| SIRT1 | si#1 5’- GCCTGATGTTCCAGAGAGA -3’ |
|  | si#2 5’- GACATGAACTATCCATCAA -3’ |
|  | si#3 5’- GGATGAAAGTGAAATTGAA -3’ |
| TET1 | si#1 5’- GCAGTATGCTCCAGTAGCT -3’ |
|  | si#2 5’- GGACTCAGATGATCTATCA -3’ |
|  | si#3 5’- GCAACACGGTGGTTTTCAA -3’ |
| PUS1 | si#1 5’- GCCAGAGCTTCATGATGCA -3’ |
|  | si#2 5’- GTCGGGTCCTCACAATTCA -3’ |
|  | si#3 5’- GGGTGCACTTCGAGAAGTA -3’ |
| TET2 | si#1 5’- GCAACATAAGCCTCATAAA -3’ |
|  | si#2 5’- GTAGCAGTGGAGAGCTACA -3’ |
|  | si#3 5’- CCAGTAAACTAGCTGCAAT -3’ |
| NSUN2 | si#1 5’- GAAGCATCGTGCTGAAGTA -3’ |
|  | si#2 5’- GGGTTATCCTCACAAATGA -3’ |
|  | si#3 5’- GCATCATGGTGGTCAACCA -3’ |
| ALKBH5 | si#1 5’- GATCGCCTGTCAGGAAACA -3’ |
|  | si#2 5’- GTCCTTCTTTAGCGACTCT -3’ |
|  | si#3 5’- GCTGCAAGTTCCAGTTCAA -3’ |
| METTL14 | si#1 5’- CAACTACAATGCAGAAACA -3’ |
|  | si#2 5’- GAAGACGCCTTCATCTATT -3’ |
|  | si#3 5’- GGACCAACGCTTACAAATA -3’ |
| FTO | si#1 5’- GTCACGAATTGCCCGAACA -3’ |
|  | si#2 5’- GACAAAGCCTAACCTACTT -3’ |
|  | si#3 5’- GAGCTTTGAGTCCTATGCT -3’ |
| DNMT2 | si#1 5’- GCGATATGCTCTTCTGTTA -3’ |
|  | si#2 5’- CTGCGATATTTCACTCCTA -3’ |
|  | si#3 5’- GTAGTAGCTAAACTAATCA -3’ |
| EP300 | si#1 5’- GCACAGAAGTGAATTCTCA -3’ |
|  | si#2 5’- GTATGAATCTGCAAACAAT -3’ |
|  | si#3 5’- GGACTGCAGTCTATCATGA -3’ |
| KAT2A | si#1 5’- GAAGCTGATTGAGCGCAAA -3’ |
|  | si#2 5’- CGAAACCACTCATGTCTTT -3’ |
|  | si#3 5’- GCCATCGGCTACTTCAAAA -3’ |
| CREBBP | si#1 5’- GCACAGCCGTTTACCATGA -3’ |
|  | si#2 5’- GCTCTATAATCGCAAGACA -3’ |
|  | si#3 5’- GGAGCCATCTAGTGCATAA -3’ |
| KDM1B | si#1 5’- GCAACTGCATAACTTTGGA -3’ |
|  | si#2 5’- GTCAATGGGTGTATTAACA -3’ |
|  | si#3 5’- GGCCTACGATATCATTGCT -3’ |
| KDM1A | si#1 5’- GCTCGACAGTTACAAAGTT -3’ |
|  | si#2 5’- GTTGGATAATCCAAAGATT -3’ |
|  | si#3 5’- GAAGCTACATCTTACCTTA -3’ |
| RNF114 | si#1 5’- CCGTGTGCTTAGAGGTGTA-3’ |
|  | si#2 5’- GGCACCGGTTTTCTTATGA-3’ |
|  | si#3 5’- GGCCACCATTAAGGATGCA-3’ |
| BARD1 | si#1 5’- GAGAGACTTTGCTCCATAT-3’ |
|  | si#2 5’- CAGTGAGCTTGCAGTAATT-3’ |
|  | si#3 5’- GACTCAGACCATCAATACA-3’ |
| RNF40 | si#1 5’- GCAAGAAGATCGCGGATGA-3’ |
|  | si#2 5’- GCATCGAGTTTGAGCAGAA-3’ |
|  | si#3 5’- GATGCCAACTTTAAGCTAA-3’ |
| PRICKLE3 | si#1 5’- GGGTCAGACTCGGAATCTT-3’ |
|  | si#2 5’- GTCGGCTAATCTCGGACTT-3’ |
|  | si#3 5’- GTCGGCTAATCTCGGACTT-3’ |
| TRAIP | si#1 5’- GAACCATTATCAATAAGCT-3’ |
|  | si#2 5’- CCAGCATGGTTACTACGAA-3’ |
|  | si#3 5’- GGAAGAACGCAATGCTACT-3’ |
| SYVN1 | si#1 5’- CCATCTTCATCAAGTATGT-3’ |
|  | si#2 5’- CCGTATGGATGTCCTTCGT-3’ |
|  | si#3 5’- TGCTGCAGATCAACCAGTA-3’ |
| MDM2 | si#1 5’- GAGAGCAATTAGTGAGACA-3’ |
|  | si#2 5’- GAGAGCAATTAGTGAGACA-3’ |
|  | si#3 5’- AGTTGAATCTCTCGACTCA-3’ |
| RNF113A | si#1 5’- GACCCACAATCCAATGATA-3’ |
|  | si#2 5’- GCGAAAGAATTGATTGCTA-3’ |
|  | si#3 5’- GGATGCAATTCCCATTACT-3’ |
| TRIM8 | si#1 5’- CCGCAAGATTCTCGTCTGT-3’ |
|  | si#2 5’- GTGGACAACTGTTACTGTT-3’ |
|  | si#3 5’- TGAACGAAGTGGCCAAGAA-3’ |
| TRIM21 | si#1 5’- GCAGCACGCTTGACAATGA-3’ |
|  | si#2 5’- GGACAATTTGGTTGTGGAA-3’ |
|  | si#3 5’- GCTTTCTGCTCAAGAATCT-3’ |
| STUB1 | si#1 5’- CTGTGAAGGCGCACTTCTT-3’ |
|  | si#2 5’- GCTCTTCGAATCGCGAAGA-3’ |
|  | si#3 5’- AGCGCTGGAACAGCATTGA-3’ |

Supplementary Table 4. Primers used in RT-qPCR

| Name | Sequence |
| --- | --- |
| METTL3 | F: TTGTCTCCAACCTTCCGTAGT |
|  | R: CCAGATCAGAGAGGTGGTGTAG |
| G6PD | F: CGAGGCCGTCACCAAGAAC |
|  | R: GTAGTGGTCGATGCGGTAGA |
| 6PGD | F: ATGGCCCAAGCTGACATCG |
|  | R: AAAGCCGTGGTCATTCATGTT |
| PGLS | F: GGAGCCTCGTCTCGATGCTA |
|  | R: GAGAGAAGATGCGTCCGGT |
| TRIM21 | 1F: TGCTTCTGAGCGGAAACTGA |
|  | 1R: ATCATTGTCAAGCGTGCTGC |
|  | 2F: GCAGCACGCTTGACAATGAT |
|  | 2R: TTGGCTAGCTGTCGATTGGG |
|  | 3F: CCCAATCGACAGCTAGCCAA |
|  | 3R: CCTAATGCCACCTGGAGCTT |
|  | 4F: CTCCAGGTGGCATTAGG |
|  | 4R: TGAGCTCCTGTAGGGC |
|  | 5F: TAGATCGAAGGTGCCACAGC |
|  | 5R: AGTATCAGCCACGGATTGGC |
|  | 6F: GAGACAAGTGAGGCTTGGAG |
|  | 6R: CTGAAGGTGGAGGGGAGTCT |
|  | 7F: GCCAAGTTGGGATTTTCCTGG |
|  | 7R: AGGTGGTTCAGAGTTCATGG |
|  | 8F: GATCCCAGCAAGCGAGC |
|  | 8R: CCATACAAGATATAATAGAGATC |
| CNT1 | F: AGGGGTCTAGCTCTTGCTG |
|  | R: ACGCAATGAATCCTGGTTCTG |
| CNT2 | F: AGGACTGACGCACAAGGAC |
|  | R: ATAGGCAGCATAGGCCAAACA |
| CNT3 | F: CACAGAGCCCTTCCTCTTTTTG |
|  | R: GCCAGAACCAATGGCTGTTTAG |
| GAPDH | F: TGCACCACCAACTGCTTAGC |
|  | R: GGCATGGACTGTGGTCATGAG |
