# Supplementary figures and images for: METTL3 confers oxaliplatin resistance through the activation of G6PD-enhanced pentose phosphate pathway in hepatocellular carcinoma
Source: Cell Death Differ. 2024 Oct 29;32(3):466–79. doi: 10.1038/s41418-024-01406-2 (PMC11894169; doi:10.1038/s41418-024-01406-2)

1D

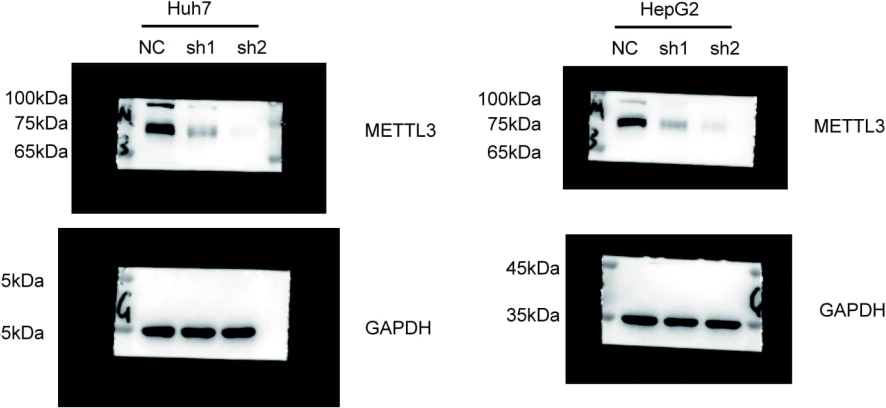

2C

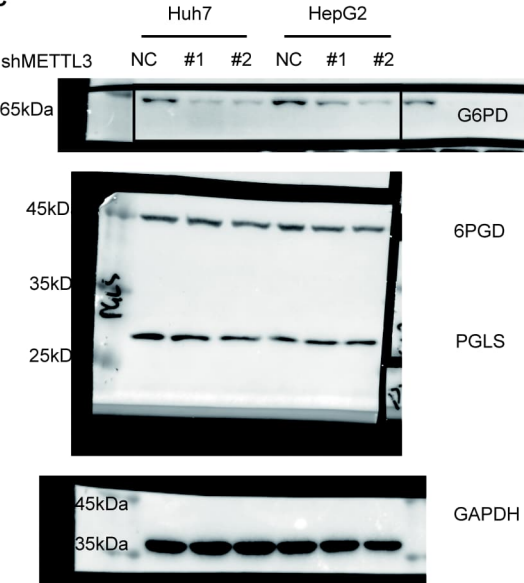

2F

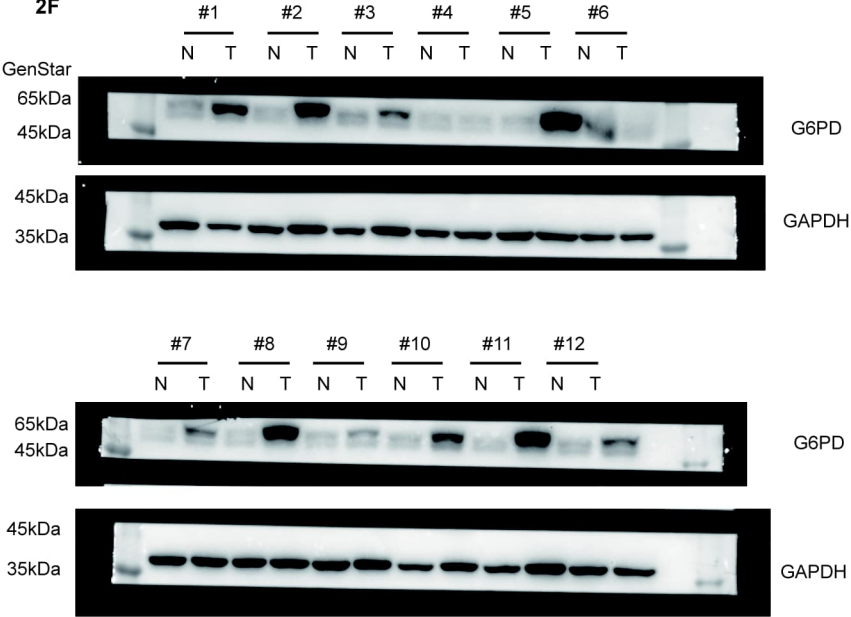

2I

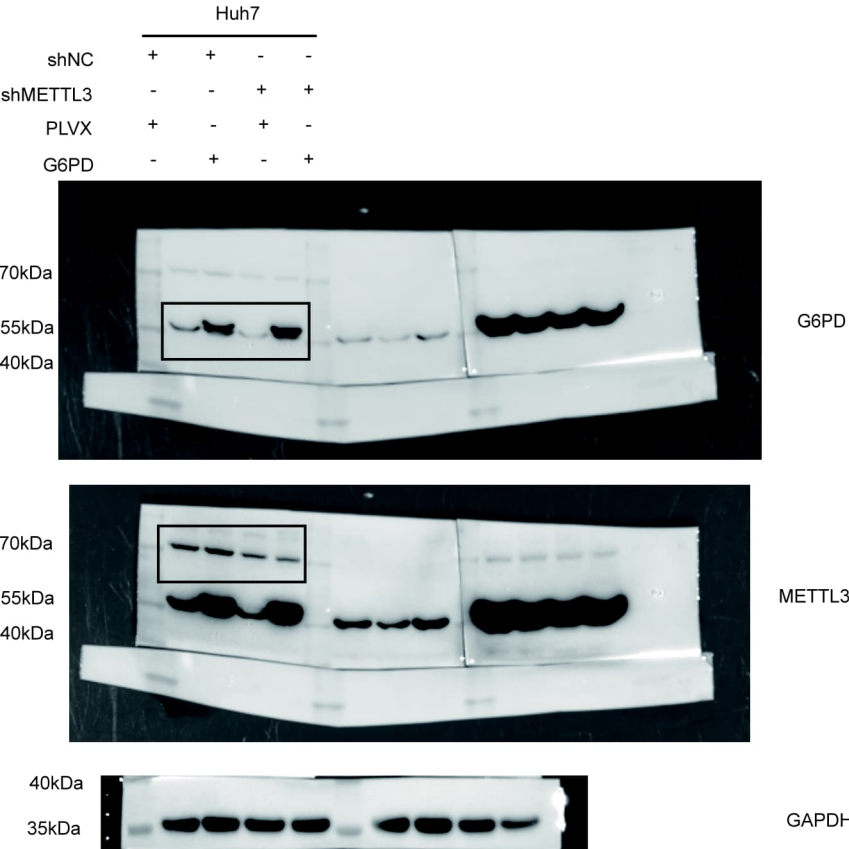

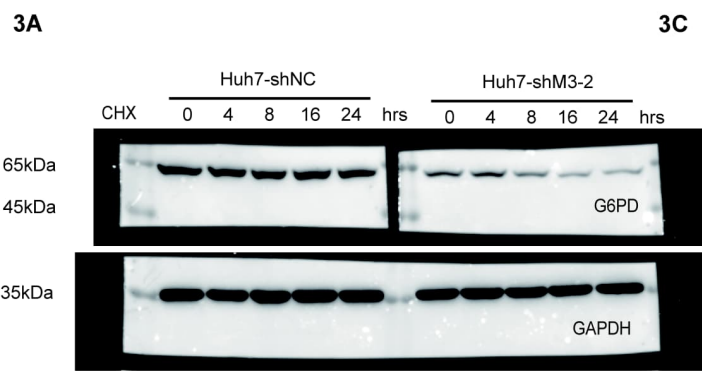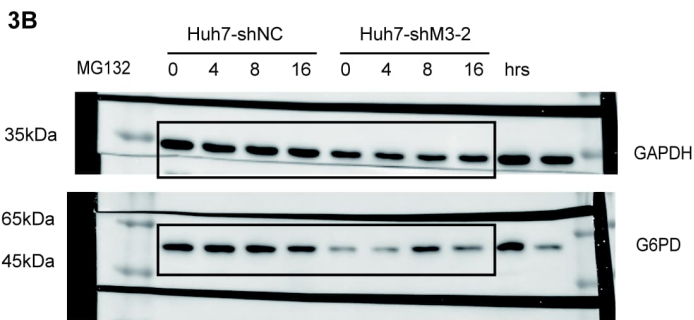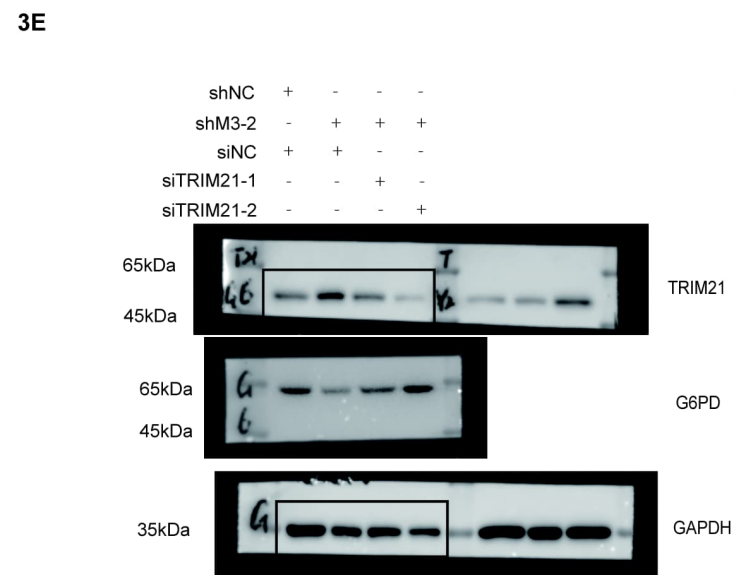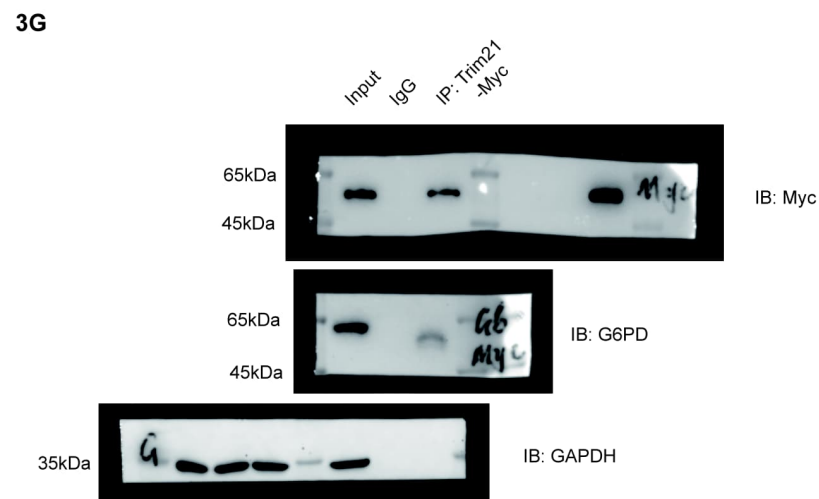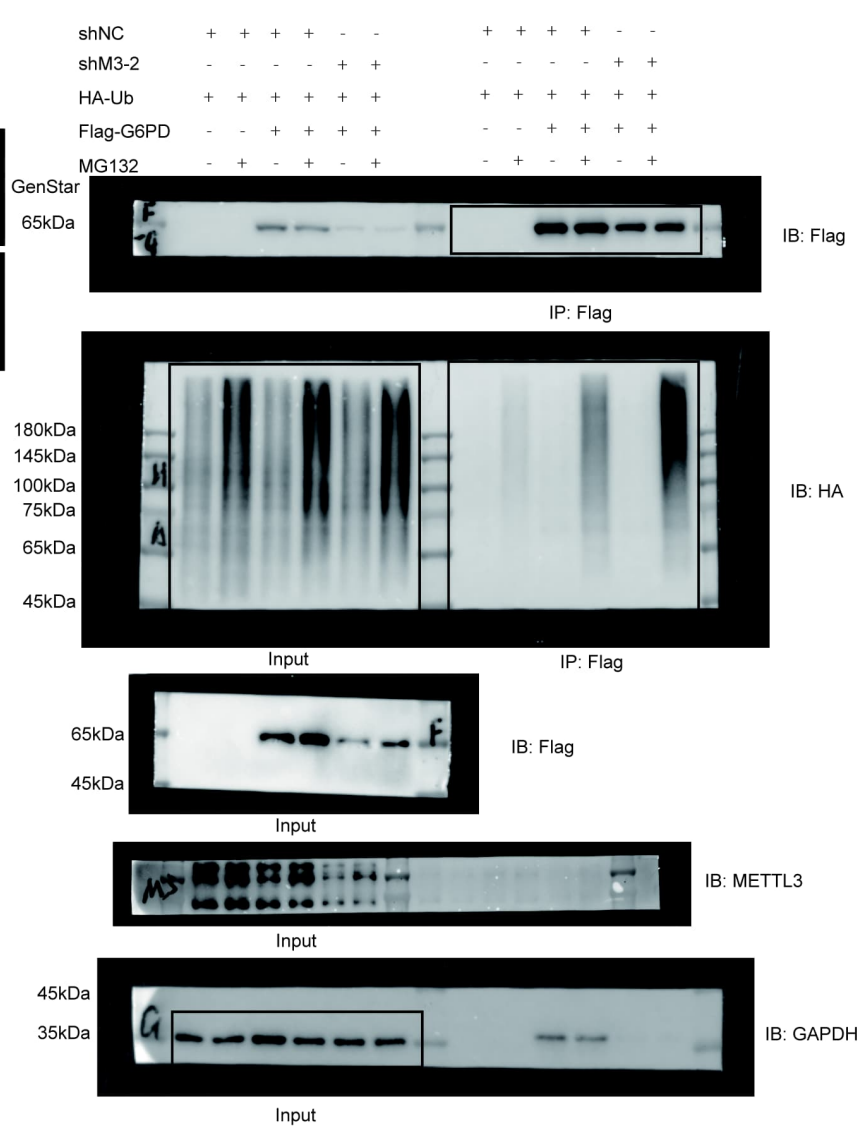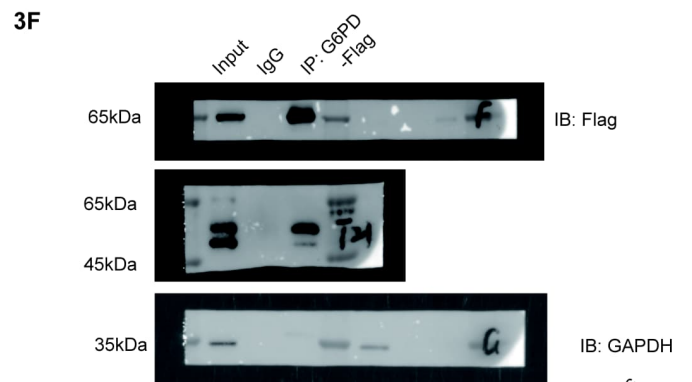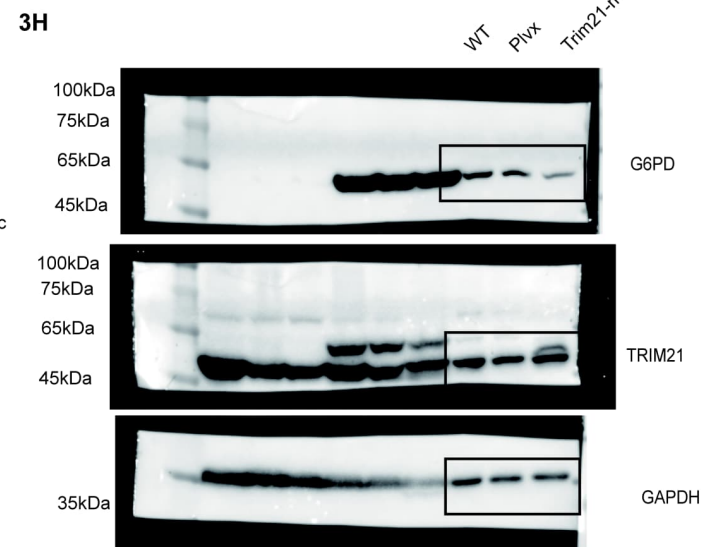

4A

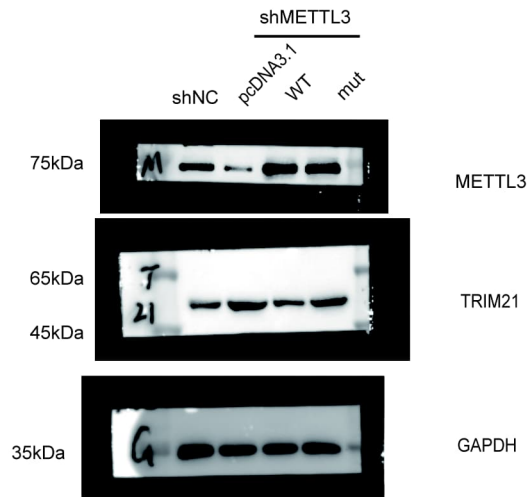

4B

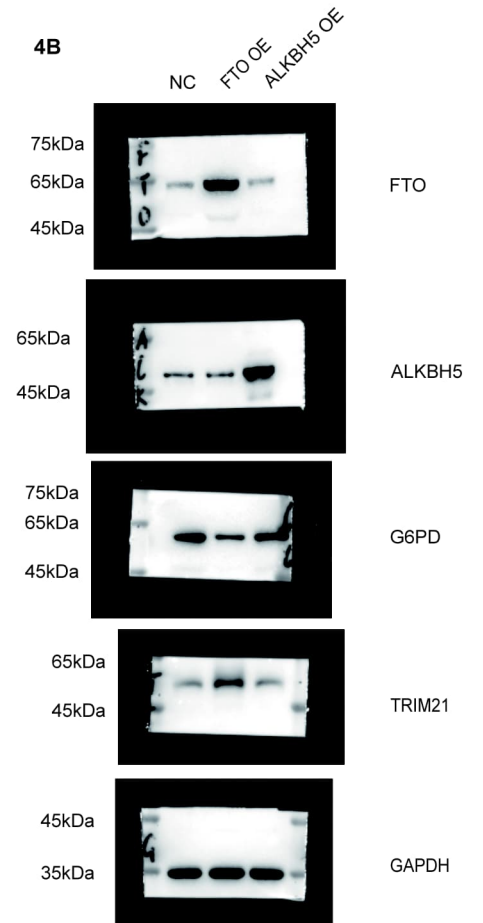

4C

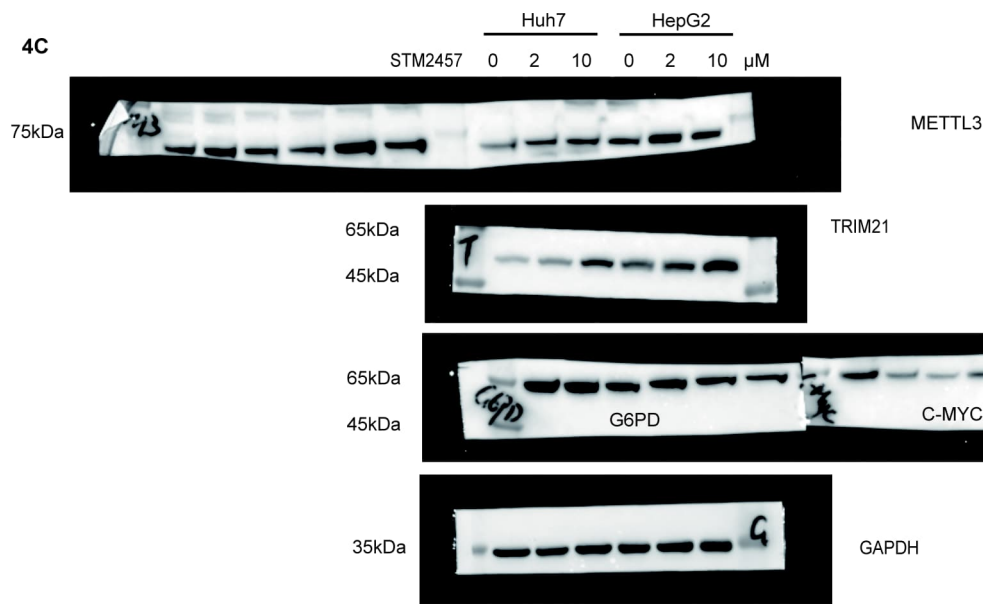

4K

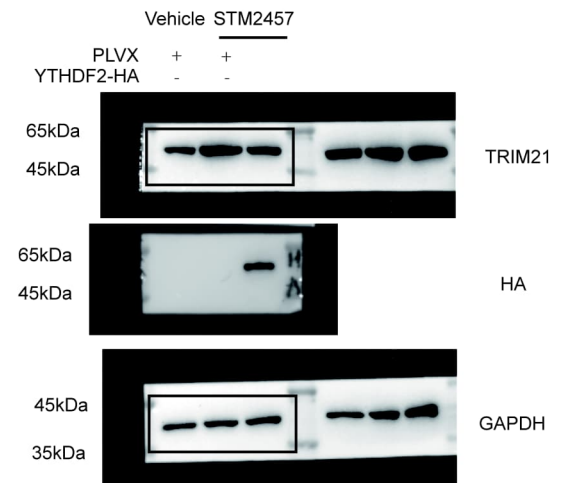

4J

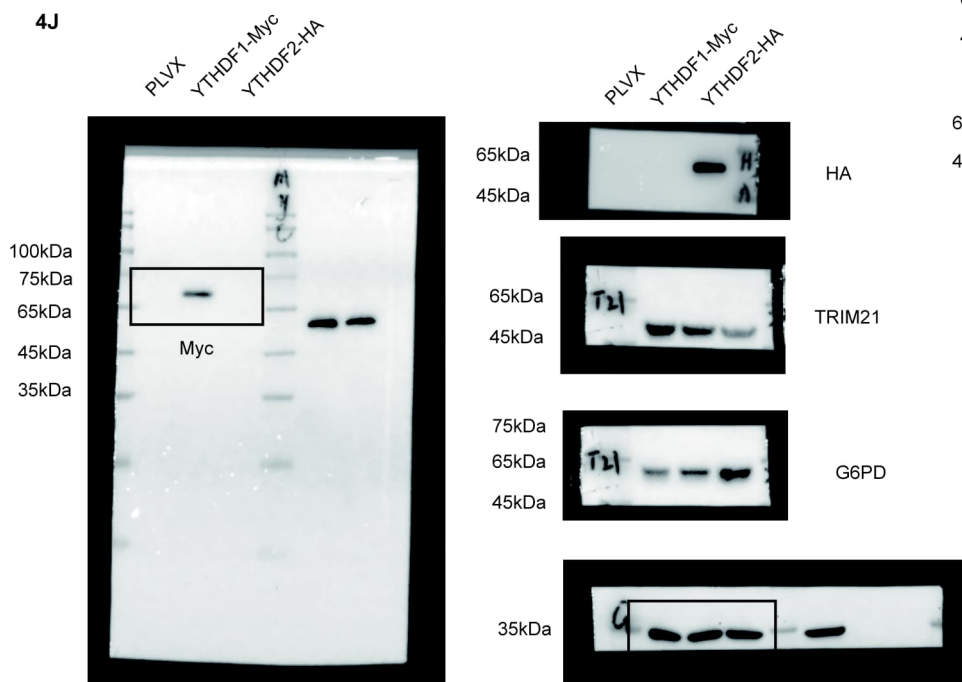

5D

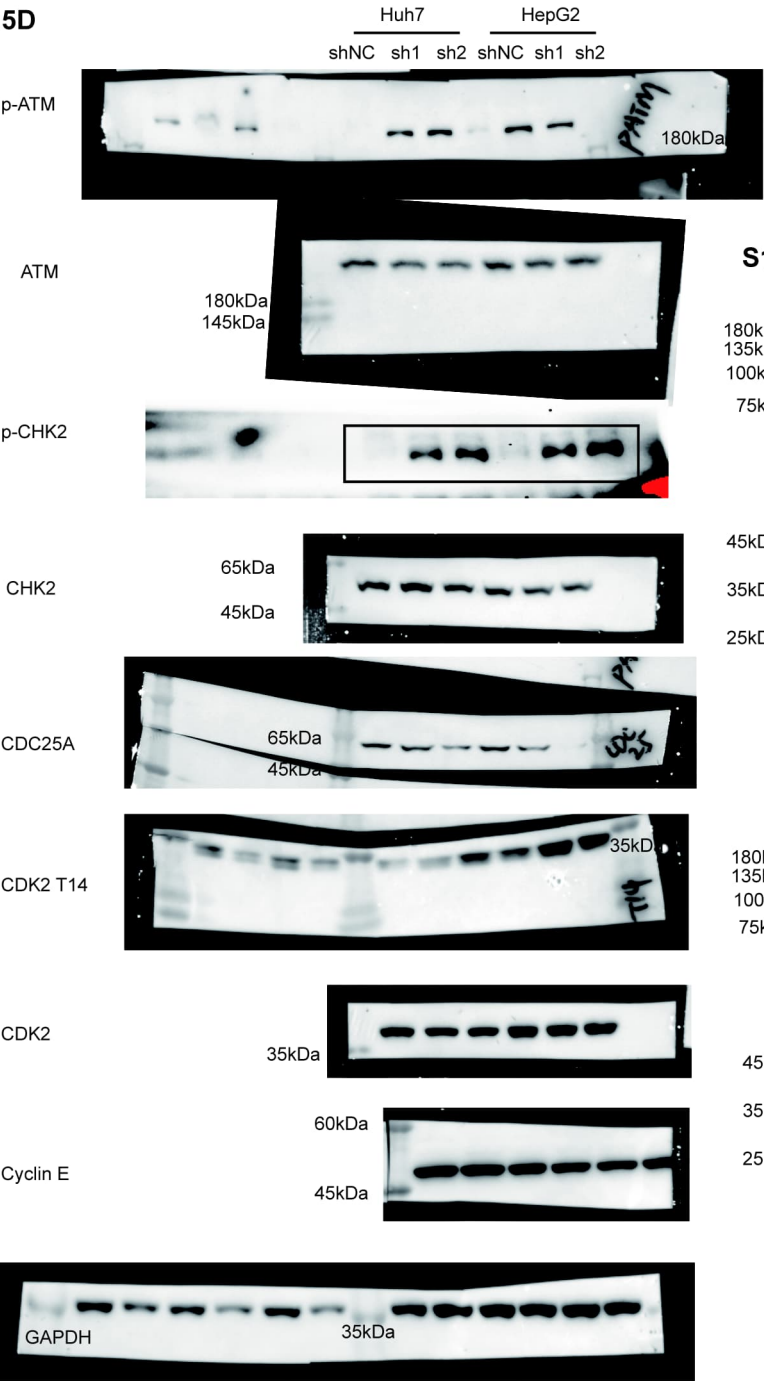

S1A

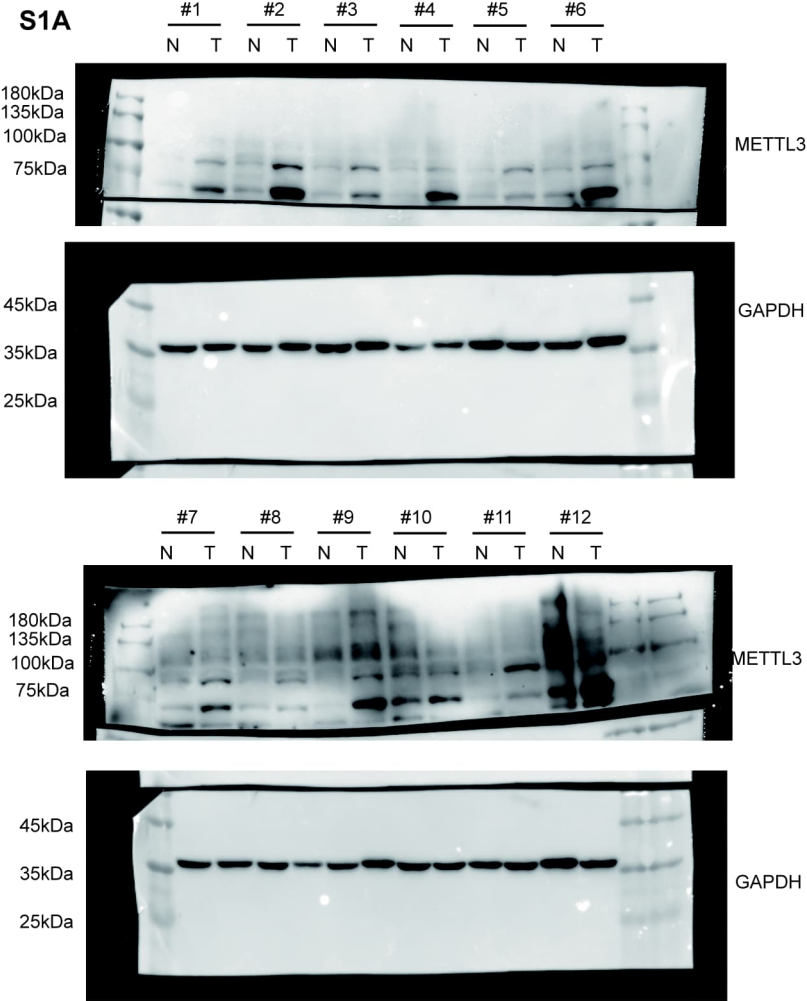

6G

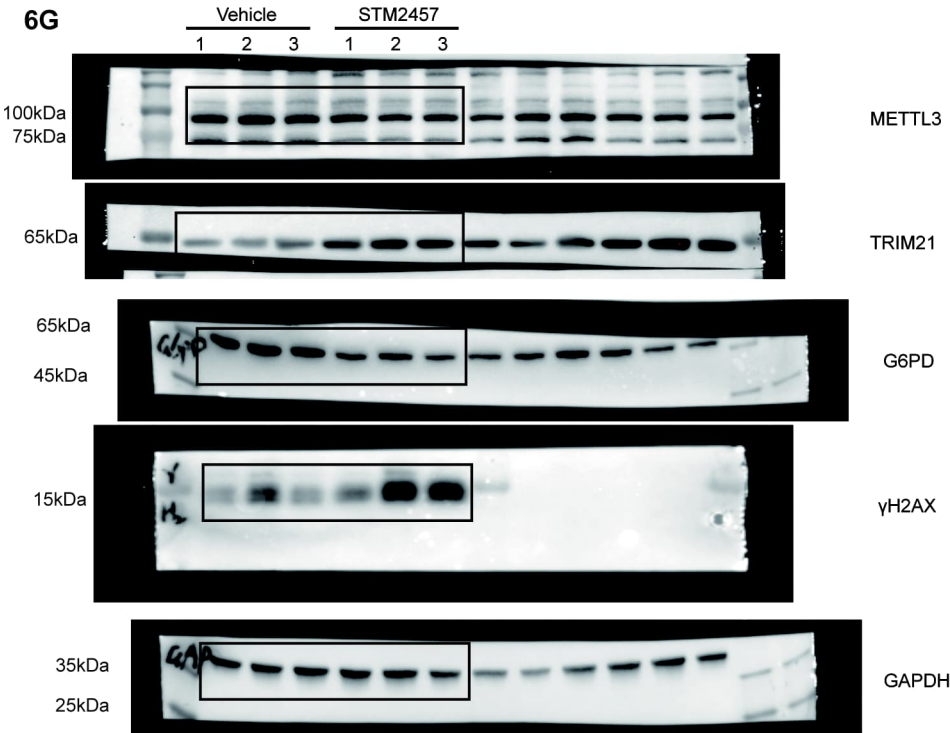

S3B

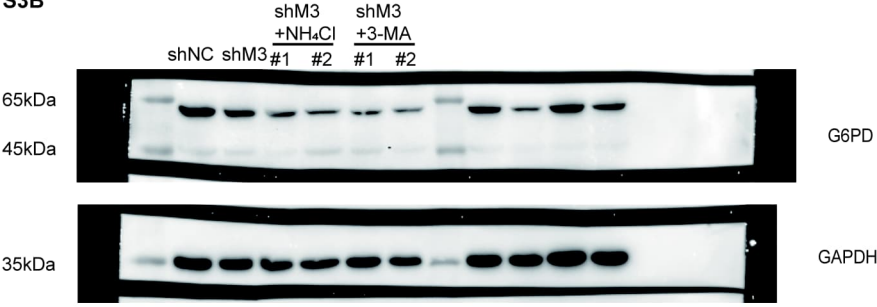

S3D

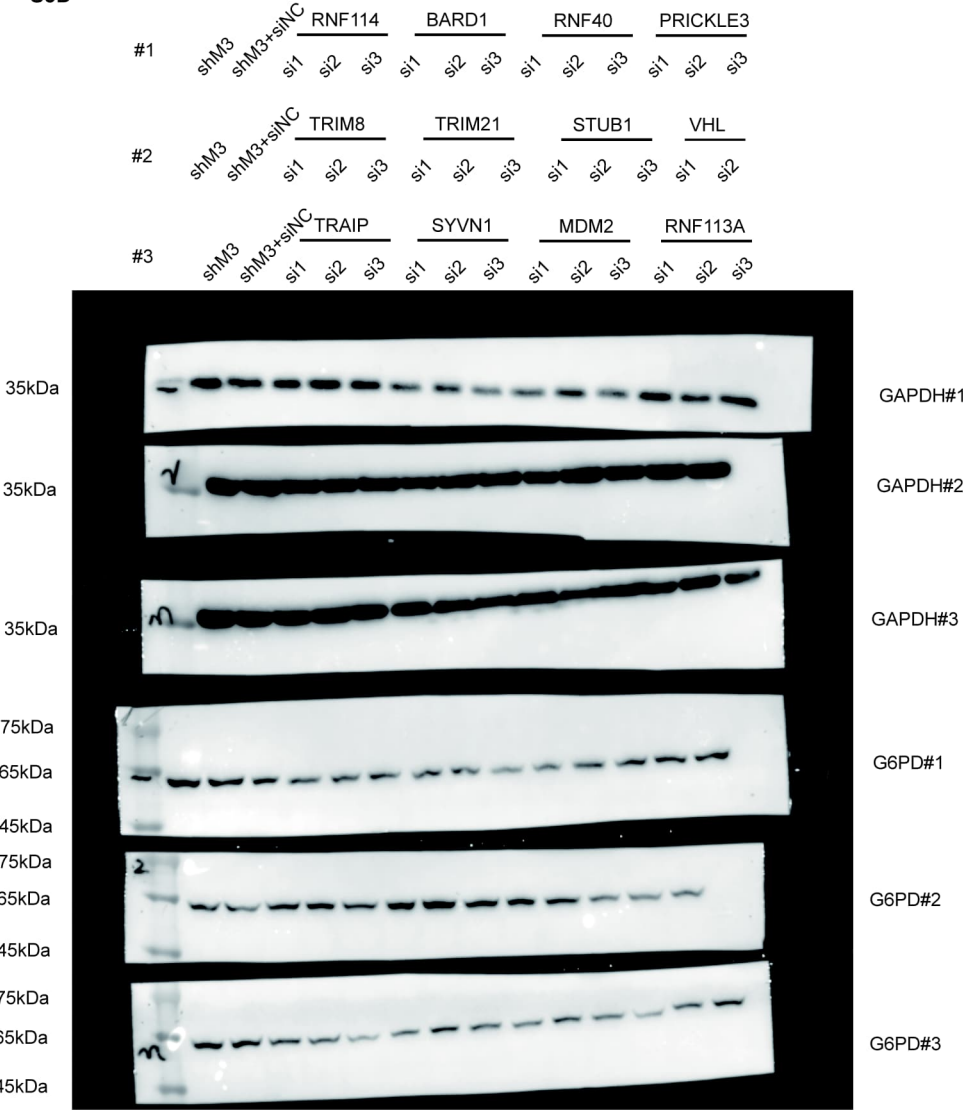

S3E

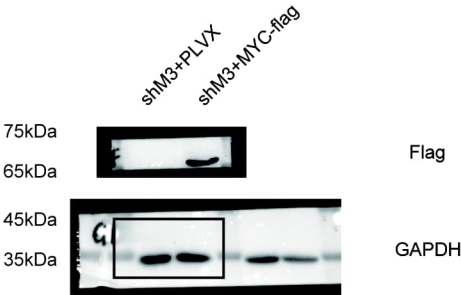

S4B

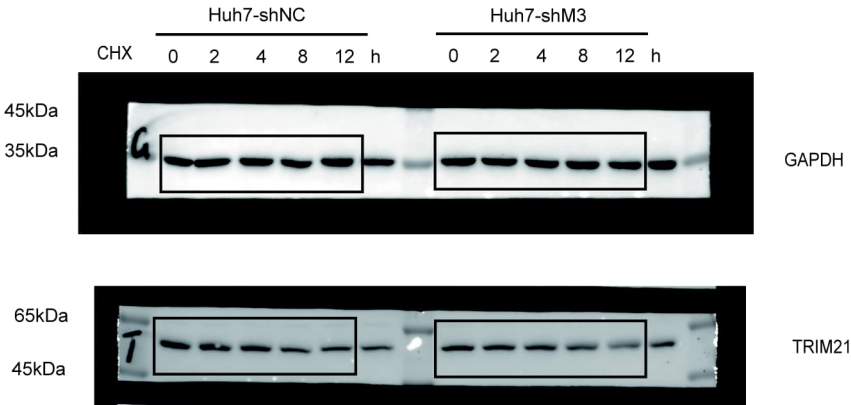

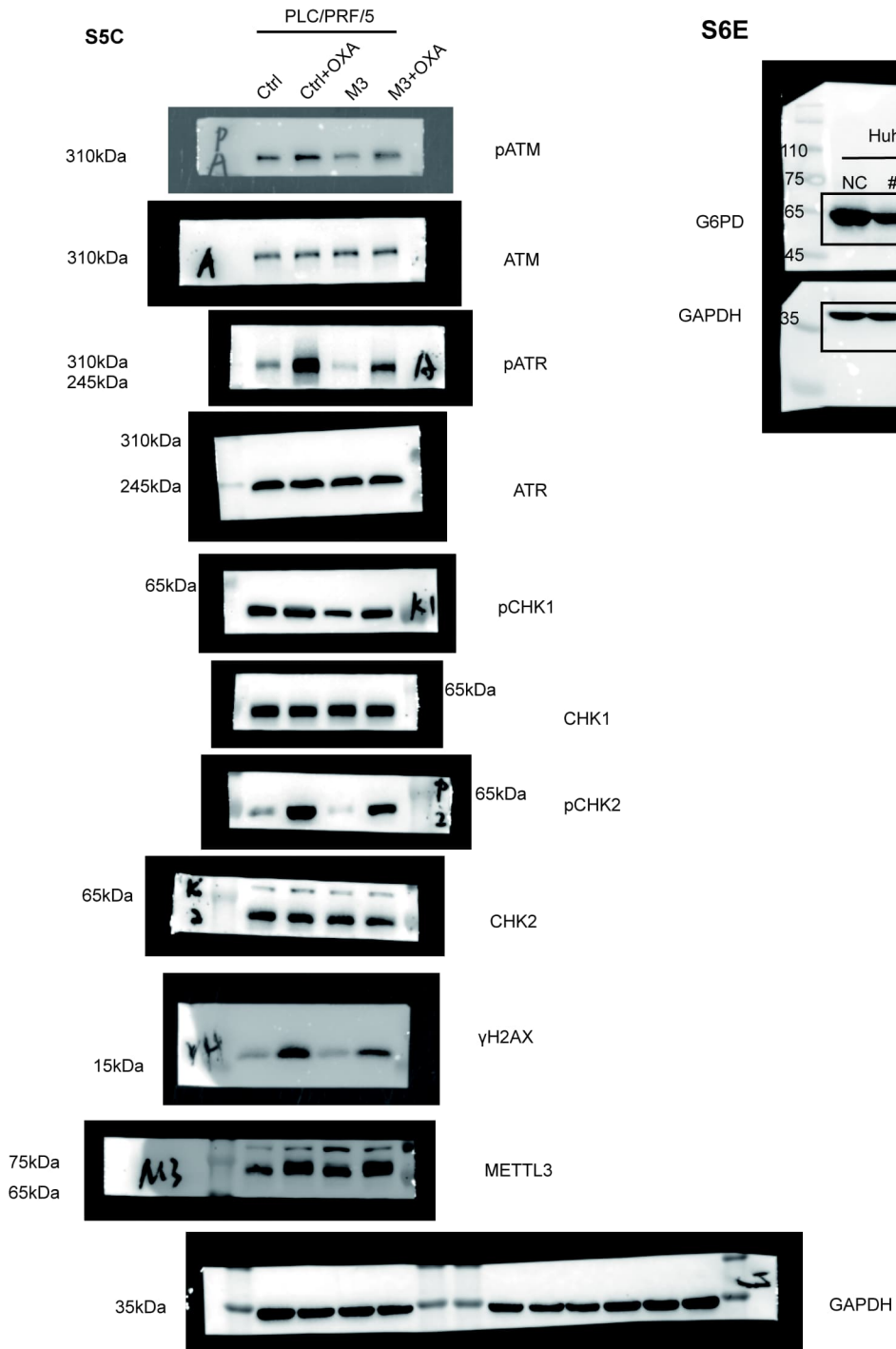

**S6E**

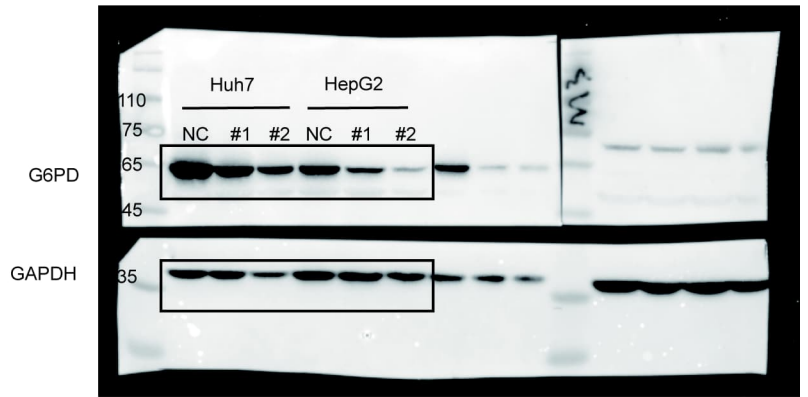

Supplement: Supplementary file 3 — Original images of WB [file 41418_2024_1406_MOESM3_ESM.pdf]
